# Carbon monoxide-driven proton respiration enables facultative anaerobes to survive electron acceptor limitation

**DOI:** 10.1093/ismejo/wrag059

**Published:** 2026-03-18

**Authors:** Yuka Adachi Katayama, Masao Inoue, Shunsuke Okamoto, Yoshihiko Sako, Ryoma Kamikawa, Chris Greening, Takashi Yoshida

**Affiliations:** Graduate School of Agriculture, Kyoto University, Kitashirakawa Oiwake-cho, Sakyo-ku, Kyoto 606-8502, Japan; Department of Microbiology, Biomedicine Discovery Institute, Monash University, Clayton, VIC 3800, Australia; Graduate School of Agriculture, Kyoto University, Kitashirakawa Oiwake-cho, Sakyo-ku, Kyoto 606-8502, Japan; Ritsumeikan Global Innovation Research Organization, Ritsumeikan University, Kusatsu, Shiga, 525-8577, Japan; College of Life Sciences, Ritsumeikan University, Kusatsu, Shiga, 525-8577, Japan; Graduate School of Agriculture, Kyoto University, Kitashirakawa Oiwake-cho, Sakyo-ku, Kyoto 606-8502, Japan; Graduate School of Agriculture, Kyoto University, Kitashirakawa Oiwake-cho, Sakyo-ku, Kyoto 606-8502, Japan; Graduate School of Agriculture, Kyoto University, Kitashirakawa Oiwake-cho, Sakyo-ku, Kyoto 606-8502, Japan; Department of Microbiology, Biomedicine Discovery Institute, Monash University, Clayton, VIC 3800, Australia; Graduate School of Agriculture, Kyoto University, Kitashirakawa Oiwake-cho, Sakyo-ku, Kyoto 606-8502, Japan

**Keywords:** carbon monoxide dehydrogenase, energy-converting hydrogenase, respiration, bioenergetics, water–gas shift reaction, hydrogenogenesis, *Parageobacillus*

## Abstract

Diverse microorganisms couple the oxidation of carbon monoxide gas (CO) to the reduction of protons, producing hydrogen gas (H_2_) using nickel-containing CO dehydrogenase/energy-converting hydrogenase (Ni-CODH/ECH). Although this process yields one of the lowest free-energy gains in biology, its physiological role at environmentally relevant CO levels remains unresolved. Here, we show that Ni-CODH/ECH functions as a survival-oriented energy conservation system that enables heterotrophic facultative anaerobes to survive electron acceptor limitation, rather than primarily supporting growth or CO detoxification. Analysis of 387 genomes of *Anoxybacillaceae* species revealed that Ni-CODH/ECH had a patchy distribution and, with one exception, was mutually exclusive with the oxygen-tolerant molybdenum-containing CODH, suggesting ecological specialization. Culture experiments using three isolates (*Parageobacillus* sp. G301, *P. thermoglucosidasius* NBRC 107763, and *Thermolongibacillus altinsuensis* B1–1) demonstrated that CO-dependent proton respiration is activated during stationary phase when exogenous electron acceptors are limiting, maintaining cell density under 25% CO, whereas no effect was observed in a Ni-CODH knockout (Δ*cooCSF*) strain. RNA-seq analysis of *Parageobacillus* sp. G301 under twelve conditions revealed that Ni-CODH/ECH genes are highly expressed (top 0.2%–1.9% of all genes) under electron acceptor-free conditions, independent of CO presence, under the predicted control of the redox-dependent transcriptional repressor Rex. Δ*cooCSF* cultures accumulated more CO than the wild-type (WT), suggesting trace CO scavenging by the WT. Together, our results redefine Ni-CODH/ECH as a redox-regulated auxiliary energy-conservation strategy that supports survival and maintenance in anaerobic energy-limited environments using two ubiquitous substrates. This work extends the carboxydovore paradigm of trace gas-based survival from aerobic to spatiotemporally variable anaerobic environments.

## Introduction

Carbon monoxide (CO) is a ubiquitous trace gas in terrestrial, marine, and host-associated environments produced through various atmospheric, biogenic, and geogenic processes [1]. This gas can be harnessed by diverse bacteria and archaea as an energy and/or carbon source [1, 2]. Because of its low standard redox potential (E^0′^ = −520 mV), CO oxidation to CO_2_ can be biologically coupled to a wide range of electron acceptors with varying redox potentials, including protons, sulfate, nitrate, and oxygen, enabling flexible energy conservation under anaerobic and aerobic conditions [3, 4]. Carbon monoxide dehydrogenases (CODH) enable microorganisms to interconvert CO and CO_2_. They fall into two structurally and phylogenetically unrelated forms: nickel-containing CODH (Ni-CODH) and molybdenum-containing CODH (Mo-CODH) [4]. These two enzyme types differ not only in their cofactor usage, but also in their physiological roles and redox partnerships [4]. Ni-CODH is oxygen-sensitive and predominantly found in anaerobic microbes, which have been mainly studied in the context of carboxydotrophic growth, including CO-dependent anaerobic respiration coupled to the reduction of electron acceptors such as protons, sulfate, or nitrate, as well as acetogenesis [3–5]. In contrast, Mo-CODH, which is oxygen-tolerant and predominantly found in aerobic microbes, functions as a respiratory enzyme that supports coupling of CO oxidation to the reduction of higher potential acceptors, such as oxygen and nitrate [6]. Recent studies have revealed that Mo-CODH primarily enables aerobic heterotrophs to act as carboxydovores, scavenging atmospheric or trace CO, thereby sustaining cellular maintenance during carbon starvation, though some microbes can also grow autotrophically using this enzyme [1, 6–8]. Thus, whereas Mo-CODH underpins a well-established carboxydovore lifestyle centered on survival and maintenance at trace CO concentrations, Ni-CODH has largely been interpreted as driving carboxydotrophic growth.

One way that anaerobes can conserve energy is by coupling CO oxidation to proton reduction, resulting in the production of CO_2_ and hydrogen gas (CO + H_2_O ⇌ H_2_ + CO_2_; i.e. CO-dependent proton respiration, hydrogenogenic CO oxidation, or water-gas shift reaction). This is achieved through the association between Ni-CODH and energy-converting group 4 [NiFe] hydrogenase (ECH; energy-converting hydrogenase) [4], containing transmembrane subunits capable of proton pumping [9], thereby generating a transmembrane proton gradient that fuels ATP synthesis [10]. This system is related to structurally characterized complexes, namely the formate hydrogenlyase (FHL) of Enterobacteriales [11] and to a lesser extent membrane-bound hydrogenases (MBH) of Thermococcales [9], that respectively couple reoxidation of endogenous formate and ferredoxin to proton reduction. It is also evolutionarily related to respiratory complex I [9]. Given the similar redox potentials of CO and H_2_, the free energy yield of CO-dependent proton respiration is one of the lowest in biology (ΔG^0′^ = −20 kJ/mol) [2], allowing the production of less than 1 mol of ATP per reaction under standard conditions [12]. Yet, hydrogenogenic CO-oxidizing activity has been reported in diverse thermophilic anaerobes and facultative anaerobes from 5 phyla, 20 genera, and 32 species as of 2020 [4]. Many of these isolates harbor CO-responsive transcriptional regulators, such as CooA, a heme-binding CO-sensing transcription factor [13], RcoM, a CO-responsive regulator containing a heme-PAS domain [14], or CorQR, a two-component regulatory system involved in CO-dependent transcriptional responses [15], which modulate Ni-CODH/ECH expression in response to environmental CO [4]. Physiologically, under high CO concentrations, CO-dependent proton respiration has been proposed to support the chemolithoautotrophic growth of carboxydotrophs [4, 16]. In addition, CO toxicity and the fast reaction kinetics of Ni-CODH [17] led to the prevailing assumption that this enzyme system acts as a CO detoxification module in CO-sensitive microbes, such as some sulfate-reducing bacteria [2, 18]. However, these hypotheses do not adequately explain the physiological role of Ni-CODH/ECH for four reasons: (i) many microorganisms harboring the Ni-CODH/ECH are heterotrophs and all isolates reported to date are capable of chemoorganotrophic growth [4]; (ii) many of them are found in environments where CO is typically detected only at trace levels (e.g. soils and sediments), yet where localized or internally produced CO may still be available; (iii) Ni-CODH is dispensable for CO resistance in various CO-tolerant microorganisms [19]; and (iv) recent studies show continual aerobic respiration using atmospheric trace gasses such as CO enhances survival of diverse aerobic bacteria [7, 20, 21]. On this basis, we hypothesized that CO-dependent anaerobic respiration may also have a role in enhancing survival especially for microbes that inhabit environments with trace CO.

Ni-CODH/ECH have been found in *Anoxybacillaceae*, a family of thermophilic, spore-forming, facultatively anaerobic heterotrophic bacteria within the phylum Bacillota, isolated from a variety of terrestrial and aquatic environments [22, 23]. Three species of this family (*P. thermoglucosidasius* NBRC 107763^T^, *Parageobacillus* sp. G301, and *T. altinsuensis* B1–1) are known to mediate hydrogenogenic CO oxidation [19, 23–25] and encode a range of respiratory machinery, including Ni-CODH/ECH, multiple [NiFe] hydrogenases, nitrate reductase, and terminal oxidases [25]. The genetic requirement of Ni-CODH/ECH for CO-dependent proton respiration was previously established in the type species *P. thermoglucosidasius* [19], a model thermophilic fermenter for industrial application [26]. Using multiple independent marker-less mutants and whole-genome sequencing, we confirmed that this system is exclusively responsible for CO-dependent proton respiration [19], providing a robust genetic platform. *Parageobacillus* sp. G301 harbors both active Ni-CODH/ECH and Mo-CODH, and CO oxidation in this strain appears to be flexibly coupled to protons, nitrate, or oxygen, depending on the available electron acceptors [25]. Although some strains have been found in relatively CO-rich volcanic settings [27], many members of *Anoxybacillaceae* are found in local soils, composts, manure, and sea sediments, where only trace levels of CO exist [22, 28]. Under such conditions, the energetic contribution of the low-energy process of CO-dependent proton respiration is unclear, especially given they, like many other hydrogenogenic CO oxidizers, are capable of using a wide range of other energy sources and electron acceptors [25, 29]. In addition, their genomes lack known CO-responsive transcriptional regulators [30], leaving the mechanisms of Ni-CODH/ECH gene expression and regulation largely unresolved.

To address this knowledge gap, we investigated the physiological roles and expression patterns of Ni-CODH/ECH in three *Anoxybacillaceae* isolates using a combination of genomics, culture-based physiological assays, and transcriptomic profiling under 12 conditions. We show that CO-dependent proton respiration is not induced by elevated CO concentration, but is selectively activated during the stationary phase in the absence of external electron acceptors, potentially supplying energy for maintenance rather than robust growth or detoxification. Our findings reveal Ni-CODH as a redox-regulated auxiliary energy-conservation system that supports survival under anaerobic, energy-limited conditions. Conceptually, this work positions CO-dependent proton respiration as an anaerobic counterpart to the Mo-CODH-mediated carboxydovore lifestyle, potentially extending our understanding of carboxydovoric trace gas-based survival strategies into anaerobic environments.

## Materials and methods

### Organisms

*Parageobacillus thermoglucosidasius* NBRC 107763^T^ (DSM 2542^T^) [28] and *Parageobacillus toebii* NBRC 107807 [31] were obtained from the Biological Resource Center of the National Institute of Technology and Evaluation (NBRC, Japan). A Δ*cooCSF* mutant strain of *P. thermoglucosidasius* was derived from the wild-type (WT) strain and maintained in the laboratory as previously described [19]. *Parageobacillus* sp. G301 [25] and *Thermolongibacillus altinsuensis* B1–1 [24] were previously isolated and preserved in our laboratory.

### Genomic analysis

Representative bacterial and archaeal genomes and their phylogenetic trees were obtained from the GTDB r226 database, comprising 306 260 genomes in total [32], to detect genomes encoding putative Ni-CODH/ECH genes (see Supplementary Information; Table S1). In addition, a total of 387 genomes classified as *Anoxybacillaceae* (Taxonomy ID: 3120669) were downloaded from the NCBI reference sequence (RefSeq) database. GTDB taxonomic classifications were assigned using GTDB-Tk v2.4.0 (release R226) [33], and genome accessions with their GTDB taxonomies are listed in Table S2. The presence of CODH and hydrogenase genes was determined using DIAMOND v2.1.12 [34] with an e-value cut-off of 1 × 10^−200^. The following protein sequences were used: clade F Ni-CODH (*Parageobacillus* sp. G301, WP_285753291.1; *Rhodospirillum rubrum*, WP_200293483.1), clade E Ni-CODH (*Thermococcus onnurineus*; WP_012571978.1), and group 4a–c hydrogenase large subunit (*Parageobacillus* sp. G301 HyfG, WP_285753305.1; *T. onnurineus* MbhL, WP_012572531.1; *R. rubrum* CooH, WP_011388069.1). The phylogenetic classifications of these enzymes were based on previous studies [4, 35–37]. The detection rates of CODH genes were calculated for each genus or species. A maximum-likelihood phylogenetic tree based on 120 concatenated single-copy core genes was obtained from the GTDB backbone tree (bac120_r226). The major clades collapsed and were labeled according to the GTDB classification. Node support values represent non-parametric bootstrap scaled from 0 to 100, as provided by GTDB. The phylogenetic tree was visualized using MEGA7 [38].

### Structural prediction

Structures of the Ni-CODH/ECH and Mo-CODH from *Parageobacillus* sp. G301 were predicted using AlphaFold 3 [39] with multi-subunit complex prediction. Input sequences are listed in Table S3. Cofactor and ligand positions were inferred based on pairwise sequence alignments with previously resolved homologous structures deposited in the Protein Data Bank (Ni-CODH: PDB 6B6X from *Desulfovibrio vulgaris*; Fd/ECH: PDB 7Z0T from *Escherichia coli*; Mo-CODH: PDB 8UEM from *Mycobacterium smegmatis*) using ChimeraX [40].

### Culture experiments

Culture experiments were performed using a minimal medium as previously described [19, 29], containing per liter: 0.3 g KCl, 0.5 g NH_4_Cl, 0.1 g KH_2_PO_4_, 0.2 g MgCl_2_·6H_2_O, 0.1 g CaCl_2_·2H_2_O, 0.03 g sodium silicate, 0.1 g NaHCO_3_, 0.5 ml trace element solution SL6 [41], and 1 ml vitamin solution [42]. The yeast extract concentration was set to 0.01%.

Pre-cultures were first grown anaerobically in minimal medium containing 15 mM pyruvate. Each preculture was then used to inoculate a separate bottle, resulting in three independently prepared cultures for each condition. In the main cultures, the strains were grown at 65°C and 100 rpm in 300 ml glass bottles containing 100 ml of medium, sealed with bromobutyl rubber stoppers and phenol resin screw caps. The headspace was filled with 200 ml of a gas mixture composed of 25% CO and 75% N_2_ (Kindgas, Kyoto, Japan). Sodium pyruvate (15 mM) served as the electron donor and carbon source. When specified, 25 mM KNO_3_ or 15% O_2_ was added to the sealed bottles to monitor growth and CO oxidation coupled with reduction of nitrate or O2, respectively. In addition, 25 mM MOPS-NaOH buffer (pH 6.8, 65°C) was added to control pH changes resulting from the fermentation products.

For the transcriptomic analysis, *Parageobacillus* sp. G301 cells were cultured in a minimal medium containing 0.1% yeast extract. Either 25 mM sodium pyruvate or 20% CO was used as the electron and/or carbon source, and 58.8 mM sodium nitrate or 80% air (O_2_) was added as indicated. The cultures were incubated at 65°C with shaking at 120 rpm. For nitrate-supplemented cultures, nitrate and nitrite concentrations in the liquid phase were determined using the Griess reaction with the NO_2_/NO_3_ Assay Kit CII (Dojindo Laboratories, Kumamoto, Japan).

Cell growth was monitored by measuring optical density at 600 nm (OD_600_) using an Ultrospec 2100 Pro spectrophotometer (Biochrom, Berlin, Germany). The headspace gas composition was analyzed using a GC-2014 gas chromatograph (Shimadzu, Kyoto, Japan) equipped with a thermal conductivity detector and a Shincarbon ST packed column (2.0 m × 3.0 mm; Shinwa Chemical Industries, Kyoto, Japan) using argon (Kindgas) as the carrier gas. The column temperature was programmed to increase from 40 to 200°C. All standard measurements were performed in triplicates. Dissolved gas concentrations were calculated based on Henry’s law and the van’t Hoff equation (65°C, 1 atm), using Henry’s constants of 1.4 × 10^−5^ for CO, 9.2 × 10^−6^ for H_2_, 6.9 × 10^−4^ for CO_2_, and 2.1 × 10^−5^ for O_2_ [43]. Statistical analyses were performed using a linear mixed-effects model for repeated measures implemented in R v4.3.3.

### Transcriptomic analysis across 12 different conditions

Cells were harvested under 12 conditions: two carbon sources (25 mM sodium pyruvate or 20% CO), three electron acceptors [O_2_, NO_3_^−^, and no exogenous acceptors (i.e. protons)], and two growth phases (logarithmic and stationary). Log-phase samples were collected when the OD_600_ reached 0.1, and stationary phase samples were taken at the first time point when no OD_600_ increase was observed. Cultures were centrifuged at 7000 × g for 5 min at 25°C, and the supernatant was discarded. Cell pellets were resuspended in 4 ml of Milli-Q water and 8 ml of RNAprotect Bacteria Reagent (Qiagen, Hilden, Germany) to stabilize the RNA and then incubated at room temperature for 5 min. Total RNA was extracted using the TRIzol Max Bacterial RNA Isolation Kit (Thermo Fisher Scientific, Waltham, MA, USA) in combination with Phasemaker Tubes (Invitrogen, Carlsbad, CA, USA), following the manufacturer’s instructions. Briefly, cell pellets were resuspended in 20 μl lysozyme solution (20 mg/ml) and incubated at 37°C for 15 min. After incubation, 200 μl of Max Bacterial Enhancement Reagent (pre-warmed to 95°C) was added, and the samples were vortexed vigorously to lyse the cells. TRIzol Reagent (1 ml) was then added, vortexed thoroughly, and transferred to a Phasemaker Tube for phase separation. DNA was removed by DNase treatment using TURBO DNase (Thermo Fisher Scientific), according to the manufacturer’s protocol. Purified RNA was further purified using the AGENCOURT RNACLEAN XP kit (Beckman Coulter, Brea, CA, USA). RNA quality was assessed using an Agilent High-Sensitivity RNA kit on an Agilent 2100 Bioanalyzer (Agilent Technologies, Santa Clara, CA, USA).

Libraries for RNA sequencing were prepared using the Stranded Total RNA Prep Ligation with Ribo-Zero Plus kit (Illumina, San Diego, CA, USA) according to the manufacturer’s instructions. Libraries were diluted to 4 nM, denatured with 0.2 N NaOH, and further diluted with HT1 hybridization buffer (Illumina) to a final concentration of 12 pM. Sequencing was performed on a MiSeq System (Illumina) using a MiSeq Reagent Kit v3 (600 cycles) (Illumina). Sequencing was conducted in duplicate for stationary-phase cells grown under oxygen or in the absence of an electron acceptor and in single replicates for the other conditions, resulting in a total of 16 sequencing runs. The RNA yield and sequencing statistics are summarized in Tables S4 and S5.

Paired-end reads were processed using fastp v0.20.1 [44] to trim adapter sequences and to remove low-quality bases. Only reads with more than 80% bases with Q scores ≥30 were retained. The reads aligned to the 5S, 16S, or 23S rRNA sequences of *Parageobacillus* sp. G301 (E-value <0.1) were removed using blastn from BLAST+ v2.10.1. Read quality was assessed using FastQC (https://www.bioinformatics.babraham.ac.uk/projects/fastqc/) and summarized using MultiQC v1.9 [45]. Read mapping and transcript quantification were performed using Salmon v1.10.1 [46] with the coding genes of strain G301 (GCF_030268025.1) as the reference. Normalization and differential expression analysis was conducted using the edgeR package v3.30.0 [47] in R. Lowly expressed genes were filtered with filterByExpr function, and library sizes were normalized using the trimmed mean of M-values (TMM) method in edgeR. Differentially expressed genes (DEGs) were identified using quasi-likelihood F-tests implemented in glmQLFit and glmQLFTest, with a false discovery rate (FDR) < 0.01 and |log_2_ fold change (log FC)| >1. Heatmaps were generated using the R package gplots [48] using log_2_(TPM +1) values. Hierarchical clustering of log_2_-transformed TPM values was performed on genes using the Pearson correlation distance and Ward’s linkage method. DEGs were visualized using volcano plots with logFC on the x-axis and log_10_ FDR on the y-axis. Genes of interest (e.g. *cooS* and *coxL*) were annotated manually. Plots were generated using the ggplot2 package in R [49]. Putative Rex-binding sites in the upstream regions of target genes were manually inspected and visualized using WebLogo v3.9.0 [50].

### Reverse transcription-quantitative polymerase chain reaction validation

To validate the transcriptomic data obtained from RNA-seq, reverse transcription-quantitative PCR (RT-qPCR) was performed for selected target genes (*cooS*, *hyfG*, *coxL*, *cydA*, *ctaC*, *narG*-1, *nuoB*, *sdhA*, *ldh*, *gyrB*, and *rpoB*). Total RNA (50 ng per reaction) extracted from three biological replicates of two distinct conditions was used as the template: (i) pyruvate-grown cells without exogenous electron acceptors in the log phase, and (ii) CO-grown cells with O_2_ in the stationary phase. The reactions were conducted using the One Step TB Green PrimeScript PLUS RT-PCR Kit (Takara, Otsu, Japan) on a Thermal Cycler Dice Real Time System III (Takara). The thermal cycling profile consisted of reverse transcription at 42°C for 5 min and 95°C for 10 s, followed by 40 cycles of PCR amplification at 95°C for 5 s and 60°C for 30 s. Each run was followed by a dissociation curve analysis and only primer pairs exhibiting a single dissociation peak were utilized. For all primer pairs, the baseline was fixed at cycles 8–12, and the fluorescence threshold was set at 20. The absence of genomic DNA contamination was verified by performing RT-qPCR without reverse transcriptase using the *rpoB* primers; no amplification was observed in these controls, whereas *rpoB* was robustly detected with an average Ct of 21.4 in the experimental samples (data not shown). Data were normalized using *gyrB* and *rpoB* as internal reference genes. Relative expression levels were determined via the ΔΔCt method, with corrections based on the individual PCR amplification efficiency (*E*) of each primer pair, which ranged from 101% to 121% (Table S6). Finally, the correlation between the corrected ΔΔCt values and the log FC values from edgeR was evaluated to assess the reliability of the RNA-seq data.

## Results

### Largely mutually exclusive distribution of nickel-containing carbon monoxide dehydrogenase and molybdenum-containing carbon monoxide dehydrogenases in *Anoxybacillaceae*

To assess the phylogenetic distribution of Ni-CODH/ECH genes, we first screened 306 260 species-level representative genomes in GlobDB release R226 [32] (Fig. S1; Table S1). Putative genes encoding Ni-CODH/ECH were sporadically detected in 179 genomes across 15 bacterial and 3 archaeal phyla, 55 orders, 107 genera, and 179 species (Fig. S1). Most of these genomes belonged to the phyla *Pseudomonadota* (42 genomes), *Bacillota* (39 genomes), *Desulfobacterota* (35 genomes), *Methanobacteriota*_B (13 genomes), and *Halobacteriota* (10 genomes). Putative genes encoding Ni-CODH/ECH are found in genera known to use a wide range of electron acceptors, spanning microaerophiles (e.g. *Campylobacter*), methanogens (e.g. *Methanoregula*), acetogens [e.g. *Neomoorella* (formerly *Moorella*)], sulfate reducers (e.g. *Desulfosporosinus*), sulfur reducers (e.g. *Thermococcus*), and iron reducers (e.g. *Geobacter*), in addition to numerous uncultivated lineages. These observations indicate that Ni-CODH/ECH is not essential but may confer adaptive advantages across diverse environmental and metabolic contexts. Putative CO-responsive transcriptional regulators (either CooA, RcoM, or CorQR) were detected adjacent to Ni-CODH/ECH loci in 99 genomes from 51 genera, including *Thermococcus* and *Geobacter* (Table S1), consistent with conventional CO-inducible regulation. However, such regulators were absent in the remaining 80 genomes from 59 genera, including *Campylobacter*, *Methanoregula*, *Neomoorella*, and *Parageobacillus* (Table S1), suggesting that Ni-CODH/ECH production in these taxa may be governed by cues other than elevated CO concentrations, or potentially by novel regulatory mechanisms that remain to be identified.

**Figure 1:**
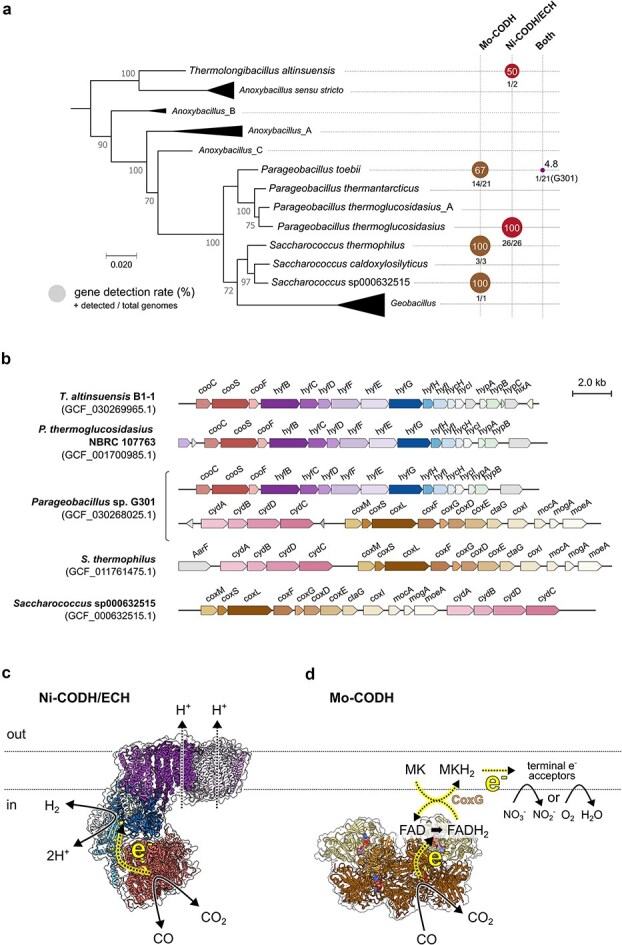
Distribution, genetic organization, and structural models of CODHs in the family *Anoxybacillaceae.* (a) A maximum-likelihood phylogenetic tree was obtained from the GTDB bacterial reference tree (bac120_r226). The tree includes representative genomes from the *Anoxybacillaceae* family, with major clades collapsed and labeled based on the GTDB classification. Node support values represent non-parametric bootstrap support scaled from 0 to 100, as provided by GTDB. Scale bar represents 0.02 substitutions per site. On the right, gene detection rates of genes encoding CODH are shown for each genus or species. Circles indicate the proportion of genomes within each clade encoding (i) Mo-CODH (brown), (ii) Ni-CODH/ECH (red), or (iii) both (purple). Gene detection rates were expressed as percentages, with circle sizes scaled accordingly. Detection rates were calculated from all available RefSeq genomes of the *Anoxybacillaceae* family. The numbers below the circles indicate the total number of genomes analyzed and the number of genomes in which each gene was detected. (b) Genetic organization of representative genomes encoding Ni-CODH/ECH and/or Mo-CODH. Gene abbreviations: *coo* (Ni-CODH subunits and a maturation factor), *hyf* (ECH hydrogenase and transmembrane antiporter-like subunits), *cox* (Mo-CODH subunits and maturation factors), and *cyd* (cytochrome ubiquinol oxidase modules). Predicted protein structure and schematic model of (c) Ni-CODH/ECH and (d) Mo-CODH from *Parageobacillus* sp. G301, generated using AlphaFold 3. Dotted lines with yellow highlights in the structures indicate the predicted electron flows. Protein backbone structures are derived from AlphaFold 3 predictions, whereas cofactor and ligand positions are predicted from previously resolved homologous structures. Arrows are schematic illustrations proposed by the authors. Prediction confidence scores and further information are provided in Figs S2 and S3. The AlphaFold 3 models are deposited in Zenodo (10.5281/zenodo.18617366).

To better understand the roles of these enzymes, we focused on *Anoxybacillaceae* lacking CO-inducible transcriptional regulators and analyzed all 387 available genomes (from 10 genera, 46 species) from RefSeq (Fig. 1a; Table S2). Genes encoding Ni-CODH/ECH were consistently found to be a combination of clade F Ni-CODH and group 4a [NiFe] hydrogenase (Hyc/Hyf type). They were detected in 28 genomes, all from the genera *Parageobacillus* and *Thermolongibacillus*, including all 26 genomes of the species *P. thermoglucosidasius* (except for two genomes annotated as *P. thermoglucosidasius*_A) and one of the two genomes of *T. altinsuensis*. In contrast, 19 *Anoxybacillaceae* within the genera *Parageobacillus* and *Saccharococcus* encode the aerobic Mo-CODH (encoded by *coxLMS*) (Fig. 1a). These two CODH systems exhibited a patchy and largely mutually exclusive distribution across the family, irrespective of phylogenetic relatedness (Fig. 1a). Only *Parageobacillus* sp. G301, classified within *P. toebii*, encodes genes for both the Ni-CODH/ECH and Mo-CODH, as reported previously [25]. The putative operons encoding both complexes have near-identical organizations across the *Anoxybacillaceae* (Fig. 1b).

Structural modeling suggests Ni-CODH/ECH and Mo-CODH generate proton-motive force in *Parageobacillus* sp. G301 through distinct respiratory architectures (Fig. 1c and d). Ni-CODH/ECH complex is predicted to form a minimalistic respiratory chain that directly couples CO oxidation to proton translocation and H_2_ production [10, 51] based on the predicted model (Fig. 1c; Fig. S2). It is formed by four interacting modules: (i) a peripheral Ni-CODH module that inputs CO-derived electrons (CooS), (ii) a ferredoxin-like protein that transfers electrons between the two catalytic enzymes (CooF), (iii) a [NiFe]-hydrogenase module that uses these electrons to reduce protons to H_2_ (HyfGHI), and (iv) a transmembrane domain that uses the energy released by electron transfer to pump protons across the membrane (HyfBCDFE). The second, third, and fourth modules are closely related to other energy-converting complexes, especially FHL [11]. In contrast, the Mo-CODH enzyme (CoxMSL) adopts a near identical structure to those described in aerobic bacterium *M. smegmatis* [52]. Our AlphaFold3 model places CoxG adjacent to the CoxMSL core near the predicted FAD-binding region (Fig. S3), suggesting it forms part of a multi-component respiratory chain, in which electrons are first transferred to quinones via CoxG and then to terminal oxidases or nitrate reductases.

### Stationary-phase hydrogenogenic carbon monoxide oxidation in three *Anoxybacillaceae* isolates

Because Ni-CODH/ECH and Mo-CODH are largely distributed in a mutually exclusive manner, we considered whether these two CODH systems might have comparable physiological functions in CO utilization. Mo-CODH enables aerobic heterotrophs to scavenge trace amounts of CO, supporting their survival over extended periods when nutrients are scarce [1, 7]. To examine this possibility, we investigated the CO oxidation and growth patterns in three thermophilic facultative anaerobes: *Parageobacillus* sp. G301 (encoding both Mo-CODH and Ni-CODH/ECH) [25], *P. thermoglucosidasius* NBRC 107763^T^ WT (encoding Ni-CODH/ECH) [28] and its Ni-CODH markerless gene disruptant (Δ*cooCSF*) [19], and *T. altinsuensis* B1–1 (encoding Ni-CODH/ECH) [24].

**Figure 2:**
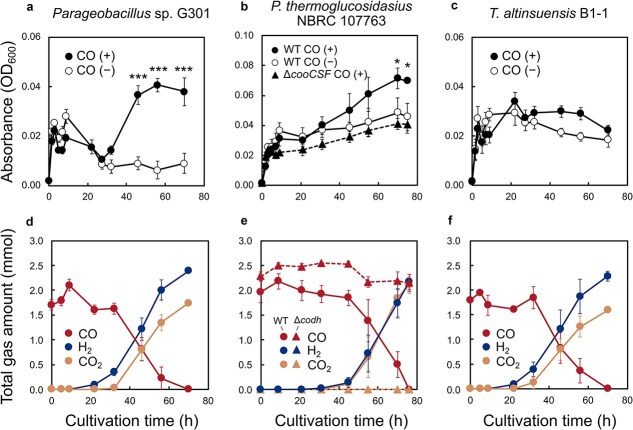
Growth of the three thermophilic facultative anaerobes from *Anoxybacillaceae* family in the presence and absence of CO. OD_600_ (black lines) of *Parageobacillus* sp. G301 (a), *P. thermoglucosidasius* NBRC 107763 WT and Δ*cooCSF* (b), and *Thermolongibacillus altinsuensis* B1–1 (c) was monitored under either 25% CO + 75% N_2_ (closed symbols) or 100% N_2_ (open symbols) in a minimal medium supplemented with 15 mM sodium pyruvate. The gas composition of the headspace of the bottles was measured in the culture of *Parageobacillus* sp. G301 (d), *P. thermoglucosidasius* WT and Δ*cooCSF* (e), and *T. altinsuensis* B1–1 (f) containing 25% CO + 75% N_2_. The figures show the total amounts of CO (red), CO_2_ (yellow), and H_2_ (blue). The experiment was performed in triplicate. The error bars represent the standard error of the mean. Statistical analysis was performed using a linear mixed-effects model for repeated measures (^***^P < .001, ^*^P < .05).

All three strains exhibited comparable growth under CO-free anaerobic conditions, reaching mean OD_600_ values of 0.028–0.049 in minimal medium containing sodium pyruvate as the organic carbon source (Fig. 2a–c). After entry into stationary phase, OD_600_ declined in G301 (from 0.016 to 0.009) and B1–1 (0.029 to 0.018) but slightly increased in *P. thermoglucosidasius* WT (from 0.036 to 0.045) under CO-free conditions (Fig. 2a–c). In the presence of 25% CO, growth proceeded similarly during the exponential phase, but after entering the stationary phase (10 h), CO supplementation altered cell density in a strain-dependent manner. In *Parageobacillus* sp. G301, OD_600_ initially decreased after 9 h (from 0.028 to 0.016), but subsequently increased significantly to 0.041 only in the presence of CO (*P* < .001), indicating CO-dependent secondary growth (Fig. 2a). *P. thermoglucosidasius* WT also exhibited a slow but significant increase under CO compared to the CO-free condition (from 0.031 at 9 h to 0.071 at 70 h; *P* < .05) (Fig. 2b). In contrast, *T. altinsuensis* B1–1 did not show a sustained increase at 70 h; however, higher OD_600_ values were observed at intermediate stationary-phase time points (45–60 h) in the presence of CO (Fig. 2c). At 70 h, all strains showed an increase in cell density, reaching OD_600_ values 4.2-, 1.5-, and 1.2-fold higher than those in CO-free conditions by 70 h in *Parageobacillus* sp. G301, *P. thermoglucosidasius* WT, and *T. altinsuensis* B1–1, respectively. In addition, the CO-dependent stationary-phase cell density enhancement was absent in the Δ*cooCSF* mutant of *P. thermoglucosidasius* (*P* < .05; Fig. 2b). Consistent with previous work using independent marker-less mutants [19], these results indicate that the observed growth enhancement is an Ni-CODH-dependent process. Gas measurements revealed that CO oxidation was negligible during exponential growth and instead initiated during stationary phase (Fig. 2d–f). The amount of CO steadily declined from 2.0–2.1 mmol to below detection limit after 21–40 h, concomitant with H_2_ accumulation and CO_2_ production to 2.2–2.4 mmol and 1.6–2.1 mmol, respectively (Fig. 2d–f). This indicates Ni-CODH/ECH-mediated hydrogenogenic CO oxidation near the expected equimolar stoichiometry.

**Figure 3:**
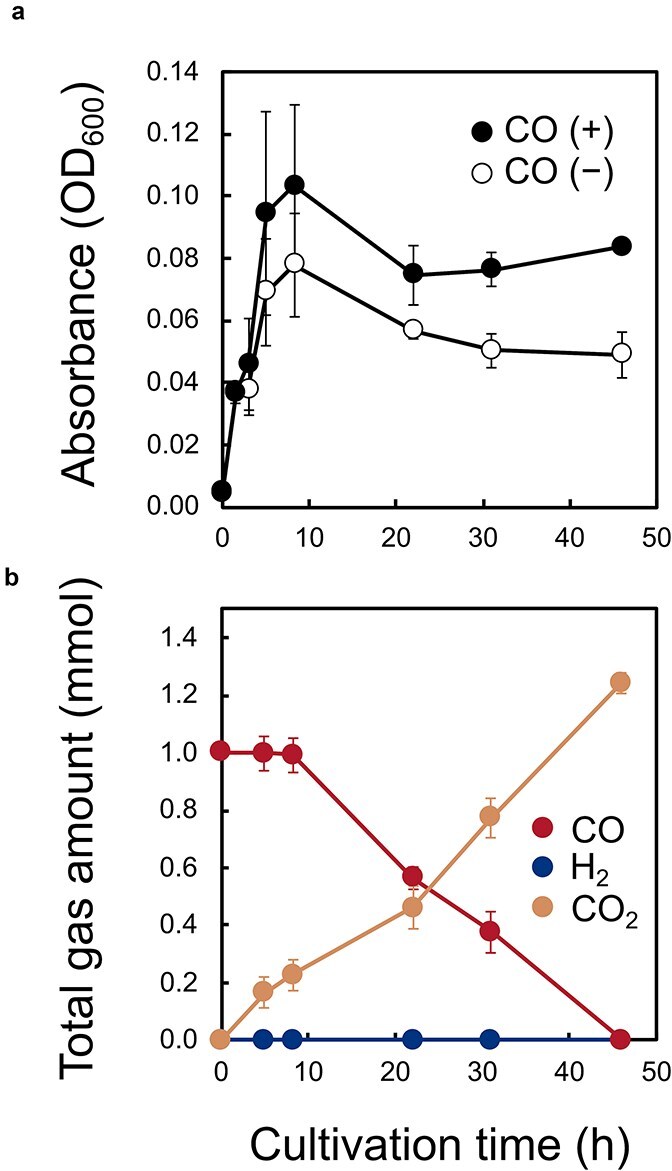
Growth and gas composition of *Parageobacillus* sp. G301 under nitrate-respiring conditions with or without CO. Cultures were grown in minimal medium supplemented with 5 mM sodium pyruvate under either 25 mM KNO_3_ in the presence of CO (closed symbols) or in its absence (open symbols). (a) Growth curves (OD_600_). (b) Total amounts of CO (red), CO_2_ (yellow), H_2_ (blue) during the same incubation. The experiment was performed in triplicate. The error bars represent the standard error of the mean.

*Parageobacillus* sp. G301 is currently the only known isolate that encodes both active Ni-CODH/ECH and Mo-CODH [25]. In nitrate-supplemented cultures, CO led to an increase in stationary-phase OD_600_ from 0.046 ± 0.015 at 5 h to 0.103 ± 0.026 at 8 h, whereas CO-free cultures reached only 0.078 ± 0.016 (Fig. 3a). In *P. thermoglucosidasius*, containing only Ni-CODH/ECH, hydrogenogenic CO oxidation of the crude lysate was completely suppressed in the presence of nitrate [29]. Consistent with this, H_2_ production was not observed during CO oxidation in *Parageobacillus* sp. G301, indicating Mo-CODH mediated CO oxidation coupled to nitrate reduction, as proposed previously [25]. Therefore, in addition to supporting aerobic survival [7], Mo-CODH might support anaerobic survival through coupling to nitrate reduction. Under O_2_-supplemented conditions, cells might have experienced a transition from aerobic to anaerobic conditions. OD_600_ became higher than in CO-free controls during the late-log to early-stationary phase (0.069 vs. 0.051 at 8 h; aerobic) but became nearly identical when CO oxidation temporarily ceased (22–31 h; putative transition phase from aerobic to anaerobic conditions) (Fig. S4a). Subsequently, when CO oxidation resumed (31–46 h; anaerobic), H_2_ was produced and OD_600_ remained stable in CO-amended cultures, whereas it declined in CO-free cultures (Fig. S4b). These observations indicated that a metabolic transition in G301 from Mo-CODH-mediated aerobic CO oxidation to Ni-CODH/ECH-mediated hydrogenogenic CO oxidation once O_2_ was depleted, providing auxiliary energy during late stationary phase.

### Nickel-containing carbon monoxide dehydrogenase expression is carbon monoxide-independent and activated under fermentative conditions

**Figure 4:**
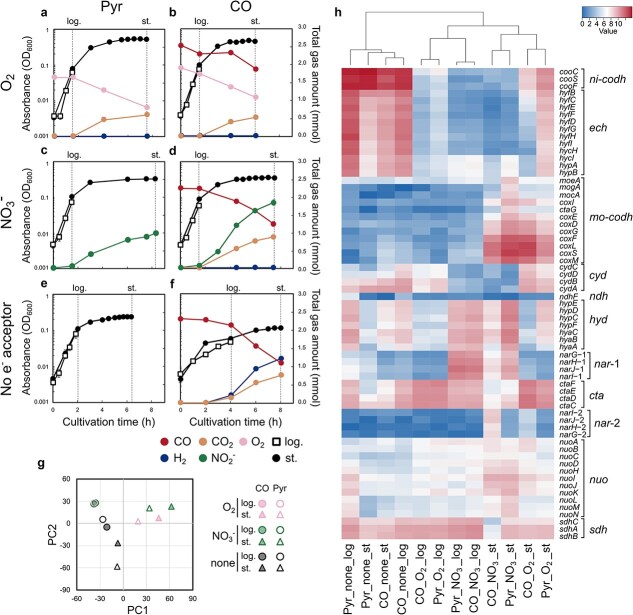
Transcriptomic analysis of *Parageobacillus* sp. G301 under twelve different carbon and electron acceptor conditions. (a–f) Growth and headspace gas composition of the strain G301 cultured under various electron acceptor conditions with either 25 mM pyruvate under 100% N_2_ (Pyr, a, c, e) or 20% CO + 80% N_2_ (CO, b, d, f) in the minimal media containing 0.1% yeast extract. OD_600_ (black circles), total gas amounts (colored circles), and cultivation time are shown. Electron acceptor conditions: (a–b) oxygen (O_2_, pink), (c–d) nitrate (NO_3_^−^, green or red), (e–f) no external electron acceptor. Open squares and black circles indicate time points of RNA sampling at logarithmic (log.) and stationary (st.) phases, respectively. The gas concentrations were monitored in the cultures harvested at the stationary phase. OD and gas measurements were performed using biological triplicate. The error bars represent the standard error of the mean. (g) PCA of transcriptomic profiles across all conditions and time points. Conditions and phases are distinguished by color and shape, respectively. (h) Heatmap showing the transcription levels (log_2_ TPM) of selected genes involved in nickel-containing carbon monoxide dehydrogenase (*ni-codh*), energy-converting hydrogenase (*ech*), molybdenum-containing carbon monoxide dehydrogenase (*mo-codh*), cytochrome ubiquinol oxidase (*cyd*), group 1d [NiFe]-hydrogenase (*hyd*), nitrate reductase (*nar*-1, *nar*-2), cytochrome *c* oxidase (*cta*), NADH dehydrogenase (*nuo, ndh*), and succinate dehydrogenase (*sdh*). Samples are clustered by hierarchical clustering (top dendrogram).

Although the culture experiments revealed that CO oxidation mediated by both Ni-CODH/ECH and Mo-CODH occurs and enhances cell density during the late-log to stationary phase, the regulatory patterns of these enzymes remain unclear. To compare the transcriptional patterns of Ni-CODH/ECH and Mo-CODH, we performed RNA-seq on *Parageobacillus* sp. G301 under 12 different conditions combining different carbon sources, electron acceptors, and growth phases (Fig. 4). Consistent with previous physiological experiments (Figs 2 and 3; Fig. S4) [25, 29], hydrogenogenic CO oxidation was evident only under electron acceptor-free conditions and H_2_ was not detected in NO_3_^−^ or O_2_-supplemented conditions (Fig. 4a–f). Principal component analysis (PCA) revealed a clear separation of transcriptomic profiles according to both electron acceptor type and growth phase, with oxygen- and nitrate-respiring conditions clustering distinctly from exogenous electron acceptor-free conditions (Fig. 4g).

Ni-CODH/ECH genes showed highly coordinated transcriptional patterns distinct from those of Mo-CODH genes. The transcription levels of all 15 Ni-CODH/ECH-related genes (*cooCSF*, QSJ37_RS02060–2070; *hyfBCEFDGHIhycHIhypAB*, QSJ37_RS02060–2130) were higher under external electron acceptor-free conditions, independent of CO supplementation, than any other tested conditions (Fig. 4h; Table S7). Under these conditions, the expression level of the catalytic subunit gene *cooS* was among the highest (top 0.2–1.9% of all genes), reaching TPM values of 2271–12 503 (Table S7). Ni-CODH/ECH genes expression also increased during the stationary phase under oxygen-supplemented conditions, with *cooS* TPM values rising from 25 and 53 at log-phase to 275 and 890 at stationary phase without and with pyruvate, respectively (|log FC| = 3.6–4.0, FDR < 0.01) (Fig. S5). In the presence of nitrate, *cooS* TPM values were consistently low (4.0–12.7) (Fig. 4h), suggesting that CO oxidation by Ni-CODH is not coupled with nitrate reduction in *Parageobacillus* sp. G301, consistent with the repression of anaerobic CO oxidation in *P. thermoglucosidasius* in the presence of nitrate (Fig. 3) [29]. G301 harbors two dissimilatory nitrate reductase complexes (NarGHJI), with two NarG genes (*narG*-1, QSJ37_RS03390 and *narG*-2, QSJ37_RS07450) coding for the catalytic alpha subunit. Among them, only *narG*-2 showed transcriptional upregulation with CO under nitrate-respiring conditions, with stationary-phase TPM values increasing from 8.5 to 240 (|log FC| = 5.5, FDR < 0.01; Fig. 3h; Fig. S5), suggesting the potential for CO responsiveness. The relative transcription level of cytochrome ubiquinol oxidase (*cydAB*, QSJ37_RS02850, QSJ37_RS02855) was low in the presence of nitrate, with TPM values of 4.3–22.2 (Fig. 4h).

Genes encoding Mo-CODH (*coxLMSG*) exhibited different expression patterns. As reported for other Mo-CODH-containing bacteria [7], Mo-CODH genes expression increased during starvation-induced stationary phase under both oxygen- and nitrate-respiring conditions, regardless of CO supplementation (Fig. 4h; Table S7). For example, *coxL* (QSJ37_RS02820) TPM values significantly increased from 6.3 and 4.2 (log-phase) to 5736 and 1232 (stationary phase) under oxygen-supplemented conditions and from 19 and 13 to 2101 and 4801 under nitrate-respiring conditions in the absence and presence of pyruvate, respectively (FDR < 0.01; Fig. S5). These patterns suggest that while both CODH systems are induced during stationary phase, Ni-CODH/ECH responds primarily to exogenous electron acceptor depletion, whereas Mo-CODH responds to starvation under respiratory conditions.

**Figure 5:**
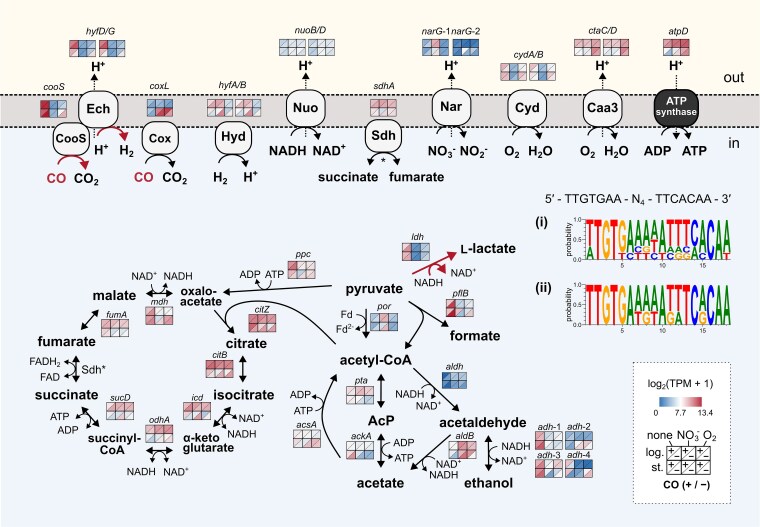
Transcriptional landscape of central metabolism and respiratory pathways under different conditions. This schematic summarizes the gene expression patterns in the key metabolic and respiratory pathways of *Parageobacillus* sp. G301 across the 12 experimental conditions described in Fig. 4. Expression levels are indicated for each gene by a heatmap showing log_2_ (TPM +1) values. Each heatmap cell indicates the following conditions: columns represent electron acceptor conditions (left: none, middle: NO_3_^−^, right: O_2_), rows indicate the growth phase (top: log-phase, bottom: stationary phase), and within each cell, the upper-left triangle corresponds to the absence of CO, and the lower-right triangle corresponds to the presence of CO. Black arrows indicate enzymatic reactions or gene products. Genes and complexes related to carbon monoxide oxidation (CooS, cox), hydrogen metabolism (Hyd, group 1d [NiFe] hydrogenase; Ech), electron transport chain (Nuo, Nar, Sdh, Cyd, Caa3, ATP synthase), TCA cycle, and mixed acid fermentation are shown. ^*^Sdh in the membrane and TCA cycle indicates the same enzyme. NAD^+^/NADH usage is putative and could be NADP^+^/NADPH. The reaction and proton translocation do not represent exact stoichiometry. Genes significantly upregulated under all four conditions lacking external electron acceptors are indicated by red arrows. Predicted rex-binding motifs were also identified upstream of redox-regulated genes. Rex is a redox-sensing transcriptional repressor that binds DNA as a homodimer when the intracellular NADH/NAD^+^ ratio is low and dissociates upon NADH binding, thereby derepressing target genes. Panel (i) shows the predicted rex-binding motif among genes previously recognized as rex-regulated in *Staphylococcus aureus* and *Bacillus subtilis*, including *ldh*, *pflB*, and *cydA*. Panel (ii) illustrates the predicted rex-binding motif upstream of Ni-CODH/ECH (*cooC*) genes across *Parageobacillus* sp. G301, *P. thermoglucosidasius*, and *T. altinsuensis* B1–1, demonstrating conserved sequence patterns consistent with the consensus motif (5′-TTGTGAA-N_4_-TTCACAA-3′). Details of the rex-binding sites are shown in Fig. S6.

Hierarchical clustering of global transcriptional profiles revealed that Ni-CODH/ECH genes clustered with genes involved in fermentative metabolism, including l-lactate dehydrogenase gene (*ldh*) and l-lactate permease gene (Fig. 5; Table S7). These genes showed significant upregulation exclusively under electron acceptor-free conditions, regardless of the carbon or electron donor source. Similar to *cooS*, *ldh* transcripts increased markedly without electron acceptors (TPM 363–1327; mean |log FC| = 4.9) (Table S7). During electron acceptor limitation, cells face the simultaneous challenge of disposing excess reductant and producing ATP. By co-expressing Ni-CODH/ECH and LDH, *Parageobacillus* sp. G301 can simultaneously dispose of NADH by producing the fermentative end-product l-lactate by LDH, while maintaining ATP production by Ni-CODH/ECH. As depicted in the metabolic reconstruction (Fig. 5), under electron acceptor limited conditions, pyruvate-grown cells also transcribe genes involved in the oxidation of pyruvate to CO_2_ via the TCA cycle, as well as those for the fermentative production of formate, ethanol, and acetate, likely to balance electron flux and energy requirements. Acetate production is likely a primary source of ATP supplemented by inputs from Ni-CODH/ECH.

Given that *ldh* is a well-characterized target of the redox-sensing transcriptional regulator Rex (or YdiH) in *Bacillus subtilis* and related Bacillota [53–57] (Fig. S6) and that the strain G301 possesses putative Rex-encoding gene (QSJ37_RS17140), we searched for conserved regulatory elements upstream of these genes. A Rex-binding-like motif (TTGTGAA-N_4_-TTCACAA) [56] was located in the upstream of previously reported Rex-regulated genes such as *cydA*, *pflB* (pyruvate-formate lyase gene), *narG*, and *ywcJ* (nitrite transporter gene), as well as 32 and 36 bp upstream of both *ldh* and *cooCSF*, respectively (Fig. 5i; Fig. S6). Furthermore, the Rex-binding-like motif was also found 37 bp and 31 bp upstream of the putative operon encoding Ni-CODH/ECH in *P. thermoglucosidasius* and *T. altinsuensis*, respectively (Fig. 5ii; Fig. S6).

To validate the reliability of the RNA-seq analysis, we performed RT-qPCR on nine selected genes (*cooS*, *hyfG*, *coxL*, *cydA*, *ctaC*, *narG*-1, *nuoB*, *sdhA*, and *ldh*). The ΔCt values, calculated using *gyrB* and *rpoB* as internal controls across three biological replicates, showed consistent expression patterns between the two growth conditions (Fig. S7a). Furthermore, a strong correlation was observed between the ΔΔCt values and the logFC values from the RNA-seq analysis (*R*^2^ = 0.96, Fig. S7b). These results confirm that the transcriptomic data reflects the physiological response of the organism under the tested conditions.

### Nickel-containing carbon monoxide dehydrogenase recycles low levels of carbon monoxide produced under anaerobic conditions

Beyond its established role in high CO conditions, Ni-CODH also contributes to the scavenging of ppm-level CO produced through biotic and abiotic processes, although effective scavenging of atmospheric trace CO has been proved only with Mo-CODH [7, 8]. We examined CO production and consumption in cultures of *Anoxybacillaceae* strains with varying CODH genotypes (Fig. S8). CO was not detected in minimal medium under anaerobic conditions (the detection limit, 0.5 ppm), whereas 2.5–5.4 ppm CO was detected in rich TGP medium for 4 h without cells (Fig. S8a), indicating abiotic formation of CO from medium components. CO levels were significantly elevated in a *P. thermoglucosidasius* mutant unable to consume CO (Δ*cooCSF*), reaching 29.5 ± 6.2 ppm after 19 h. These results suggest both abiotic and potentially biotic CO generation occurs under anaerobic conditions. In contrast, CO concentrations in *P. thermoglucosidasius* WT, *Parageobacillus* sp. G301, *P. toebii* NBRC 107807, and *T. altinsuensis* B1–1 cultures remained similar to the control. This suggests that Ni-CODH and Mo-CODH reoxidized the environmentally relevant ppm-level CO produced or these microbes do not produce trace CO as a by-product under anaerobic conditions.

## Discussion

This study identifies a previously unrecognized physiological role of anaerobic CO oxidation in thermophilic facultative anaerobes. CO-dependent proton respiration using Ni-CODH/ECH is selectively activated during the stationary phase under external electron acceptor limitation in *Anoxybacillaceae*. While energy flux from CO might be allocated to biomass synthesis if CO exists at high concentrations, our results indicate that this minimal respiration primarily supports cellular maintenance at environmentally relevant CO concentrations. This role contrasts with the classical view of CO-dependent metabolisms, which has largely emphasized CO-driven growth in anaerobic extremophiles under high CO availability. Activation of CO oxidation under energy-depleted conditions parallels the well-established role of atmospheric CO and H_2_ oxidation, the former mediated by Mo-CODH [1, 6, 7, 58]. Within *Anoxybacillaceae*, Ni-CODH/ECH and Mo-CODH exhibited largely mutually exclusive distributions (Fig. 1, Fig. S1), suggesting that these systems may support analogous physiological functions under anaerobic and aerobic conditions, respectively. In *Parageobacillus* sp. G301, CO is consistently utilized during stationary phase across multiple respiratory modes (proton, nitrate, and aerobic respiration) through both Ni-CODH and Mo-CODH (Figs 2 and 3), indicating its role as a consistent auxiliary energy source during energy deprivation. Therefore, we propose that Ni-CODH/ECH performs a function analogous to that of Mo-CODH, but is specifically adapted to strictly anaerobic, low-redox environments where high-potential electron acceptors are unavailable. Although CO-dependent proton reduction yields substantially less energy (ΔG^0′^ = −20 kJ/mol) than CO oxidation coupled to O_2_ (ΔG^0′^ = −258 kJ/mol) or nitrate (ΔG^0′^ = −181 kJ/mol), it may nonetheless provide a dependable means to generate a proton-motive force using cellular protons as the electron acceptor and endogenous or environmentally produced CO as an electron donor.

Transcriptional patterns further indicate that Ni-CODH/ECH production responds to intracellular redox balance rather than external CO availability. During the stationary phase under electron acceptor-free conditions, all three tested isolates initiated hydrogenogenic CO oxidation and completely consumed 25% CO within 80 h (Fig. 2), demonstrating a conserved physiological response across these strains. In *Parageobacillus* sp. G301, genes encoding Ni-CODH/ECH were strongly transcribed under electron acceptor-free conditions, reaching up to 0.2–1.9% of all transcripts, and this expression pattern closely followed that of *ldh*. In *B. subtilis* and related Bacillota, *ldh* expression is controlled by the redox-sensing repressor Rex, which responds to elevated NADH/NAD^+^ ratios [53–57]. The G301 genome also contains a Rex-binding motif upstream of both *ldh* and the putative operon encoding Ni-CODH/ECH (Fig. S6), identical to the Rex consensus sequence [56], suggesting that Ni-CODH/ECH production in this strain is regulated by intracellular redox status. A Rex-binding motif is also present upstream of the genes encoding Ni-CODH/ECH in *Calderihabitans maritimus* [59], suggesting that this redox-dependent regulation is potentially conserved among other hydrogenogenic CO oxidizers. During fermentation, cells must balance ATP generation with NAD^+^ regeneration, often at the cost of reduced energy yield. Under these conditions, hydrogenogenic CO oxidation can supply an auxiliary energy. As a potential single enzyme complex (Fig. 1c), this system enables proton-motive force generation either directly or via a diffusible ferredoxin pool, independently of redox carriers (e.g. quinones, NAD) and solely produces diffusible gaseous end-products, conferring resilience under fluctuating cellular redox state and metabolite levels.

Another key question is the source of CO for these bacteria. The ecological role of Mo-CODH has been well established in aerobic environments, where it enables heterotrophs to scavenge atmospheric CO and thereby sustain maintenance metabolism [7]. However, trace CO is not restricted to oxic settings and can originate from thermal, photochemical or radiolytic reactions, as well as microbial metabolism in anaerobic environments [60]. In volcanic soils or sun-exposed soils, temperatures can reach thermophilic ranges [61], where abiotic production of CO enhances as a result of thermal degradation of organic matter [60]. Field-based measurements indicate that CO is available in various anaerobic environments, including salt marshes (+0.8 to 3.6 nmol m^−2^ s^−1^ in anaerobic slurries) [62], tropical rainforest soils (−0.2 to +3.4 nmol m^−2^ s^−1^) [63], peat land (−0.3 to 0.4 nmol m^−2^ s^−1^) [64, 65], composts (+6.0 to 688 nmol m^−2^ s^−1^) [66], and hydrothermal fluids (1.9 to 5.8 μM) [67]. Consistent with these environmental observations, trace amounts of CO accumulated during anaerobic incubation of rich medium at 65°C (Fig. S8), indicating that thermal or chemical decomposition of medium components generates CO. *P. thermoglucosidasius* Δ*cooCSF* accumulated significantly more CO than the WT strain, implying that the WT strain re-oxidized endogenously produced CO. These findings indicate that thermophiles in anaerobic environments may utilize trace CO derived from a combination of local abiotic processes and metabolic byproducts. Indeed, anaerobic incubation of soils and sediments with CO resulted in H_2_ production, implying the activity of indigenous hydrogenogenic CO oxidizers [68, 69]. Furthermore, hydrogenogenic CO oxidizers have been isolated or detected in various environmental samples, such as marine sediments [70–72] and freshwater lake sediments [24, 73], and CO oxidizers have also been detected in the gut microbiome [74]. Thus, analogous to Mo-CODH-mediated oxidation of atmospheric CO in aerobic systems, Ni-CODH/ECH may enable anaerobic microbes to scavenge even trace amounts of CO, aiding cell maintenance under otherwise energy-starved conditions.

Altogether, this study redefines CO-dependent proton respiration as a low-energy survival strategy rather than a CO-responsive growth and/or detoxification process [3, 4, 18]. Our findings demonstrate that Ni-CODH/ECH is activated not by elevated CO concentrations, but by external electron acceptor limitation, enabling microbes to exploit trace levels of CO in environments that are generally low in CO such as soils and sediments. At these concentrations, CO-dependent proton respiration might not support net growth but instead provides energy to survive under energetic constraints. In this respect, this study extends the carboxydovore paradigm of survival-oriented trace CO oxidation for aerobic heterotrophs [7], into anaerobic, low-redox environments. Together, our findings expand the ecological relevance of hydrogenogenic CO oxidation from localized high-CO systems to widespread, energy-limited anaerobic ecosystems, where it provides a minimal and dependable metabolic lifeline during periods of electron acceptor limitation.

## Supplementary Material

Supplementary_Information_Katayama_v6_wrag059

Supplementary_tables_Katayama-etal_v5_wrag059

## Data Availability

The raw reads for RNA-seq were deposited in the NCBI/ENA/DDJB Sequence Read Archive under BioProject accession number PRJDB37689. The AlphaFold 3-predicted structures were deposited in Zenodo (doi:10.5281/zenodo.18617366).
