# Supplementary material for: Carbon monoxide-driven proton respiration enables facultative anaerobes to survive electron acceptor limitation
Source: ISME J. 2026 Mar 18;20(1):wrag059. doi: 10.1093/ismejo/wrag059 (PMC13077300; doi:10.1093/ismejo/wrag059)
Supplement: Supplementary_Information_Katayama_v6_wrag059 [file supplementary_information_katayama_v6_wrag059.docx]

**Supplementary Information for:
Carbon monoxide-driven proton respiration enables facultative anaerobes
to survive electron acceptor limitation**

**Yuka Adachi Katayama^1,2,*^, Masao Inoue^1,3,4^, Shunsuke Okamoto^1^, Yoshihiko Sako^1^, Ryoma Kamikawa^1^, Chris Greening^2^, Takashi Yoshida^1^**

^1^Graduate School of Agriculture, Kyoto University, Kitashirakawa Oiwake-cho, Sakyo-ku, Kyoto 606-8502, Japan; ^2^Department of Microbiology, Biomedicine Discovery Institute, Monash University, Clayton, VIC 3800, Australia; ^3^R-GIRO, Ritsumeikan University, Kusatsu, Shiga, Japan; ^4^College of Life Sciences, Ritsumeikan University, Kusatsu, Shiga, Japan

**Supplementary methods and materials**

**Global detection of CO dehydrogenases and associated [NiFe] hydrogenases**

The phylogenetic distribution of CODHs and group 4 [NiFe] hydrogenases was analyzed across 306,260 representative bacterial and archaeal genomes from the GlobDB r226 database (1). The archaeal and bacterial backbone trees were obtained from the GTDB r226 release. Protein sequence data were downloaded as species-level representative FASTA files and concatenated into a single searchable database. Putative CooS were identified using the Greening laboratory in-house database and DIAMOND blastp (v2.1.8) (2). All hits were filtered to retain only those with either query or subject coverage ≥80% and alignment length ≥75 amino acids. Additional filtering was applied by functional category using minimum amino acid identity thresholds of 50%. To identify potential Ni-CODH/ECH complexes, genomes were additionally screened for Group 4 [NiFe] hydrogenase genes using sequences in HydDB (3). A genome was classified as encoding a putative CODH/ECH complex when at least one [NiFe] Group 4 hydrogenase gene occurred within 15 genes upstream or downstream of a CooS homolog on the same contig. Distances between CooS and [NiFe] hydrogenase loci were computed from locus tags, and gene pairs were enumerated per genome. For genomes encoding putative CODH/ECH complexes, protein annotations for genes located within 10 genes of the CODH/ECH were extracted from the GlobDB r226 protein annotation files. These local gene neighborhoods were screened for the presence of putative CO-responsive transcriptional regulators (CorQR, RcoM, and CooA) based on KEGG Orthology assignments (K02019, K21696, and K21555, respectively). The resulting dataset was used for visualization of putative Ni-CODH/ECH genes distributions across microbial lineages. The archaeal and bacterial backbone trees from the GlobDB r226 release were visualized using R (v4.3.1) with the packages ggtree (4), treeio (5), ape (6), ggtreeExtra (7), cowplot (8), and colorspace (9). Phylum-level taxonomy was extracted from the GlobDB taxonomy file and visualized as colored outer rings.

**Trace CO detection assays**

To assess trace level of CO, five *Anoxybacillaceae* strains (*P. toebii*, *Parageobacillus* sp. G301, *T. altinsuensis*, *P. thermoglucosidasius* wild-type and Δ*cooCSF* strains) were cultured in TGP medium (10), which contained per liter: 17 g tryptone, 3 g soy peptone, 5 g NaCl, 2.5 g K_2_HPO_4_, 4 mL glycerol, 4 g sodium pyruvate. Cultures were incubated at 65°C and 100 rpm in a N_2_ headspace (100%). Each 300 mL serum bottle contained 50 mL of liquid medium and 250 mL of headspace. After 4 h of incubation, the CO concentrations in the headspace were measured using CO detector tubes (1LC; Gastec Co., Kanagawa, Japan). For time-course measurements, *P. thermoglucosidasius* wild-type and Δ*cooCSF* strains, and *Parageobacillus* sp. G301 cells were cultured under the same conditions and headspace CO concentrations were measured at 0, 4, 19, and 25 h using the same CO detector tubes. As the CO detectors required opening the bottles for measurement, different bottles were used at each time point. All experiments were performed in triplicates.

**Supplementary figures**

**
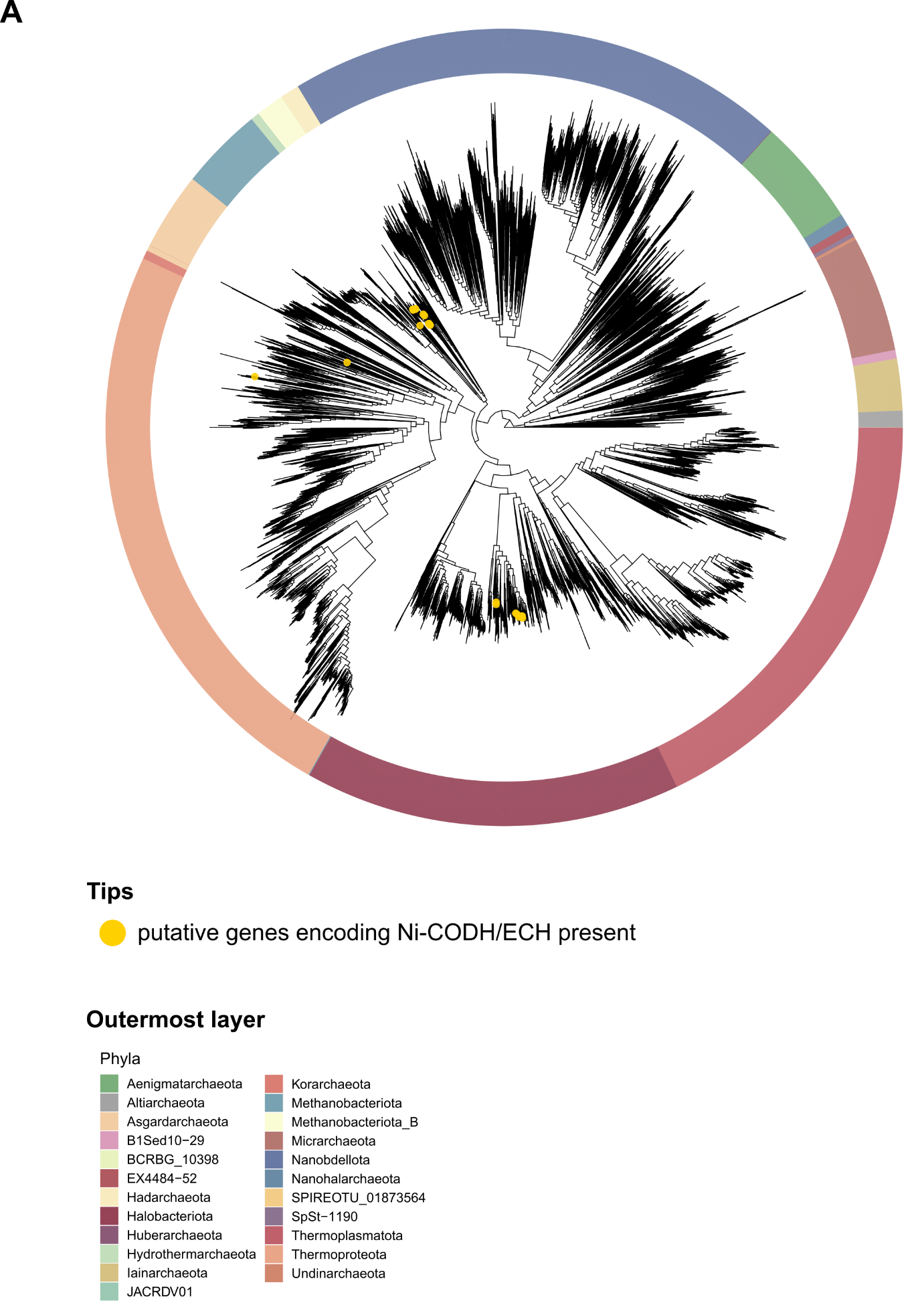
**

(legend on the next page)

**
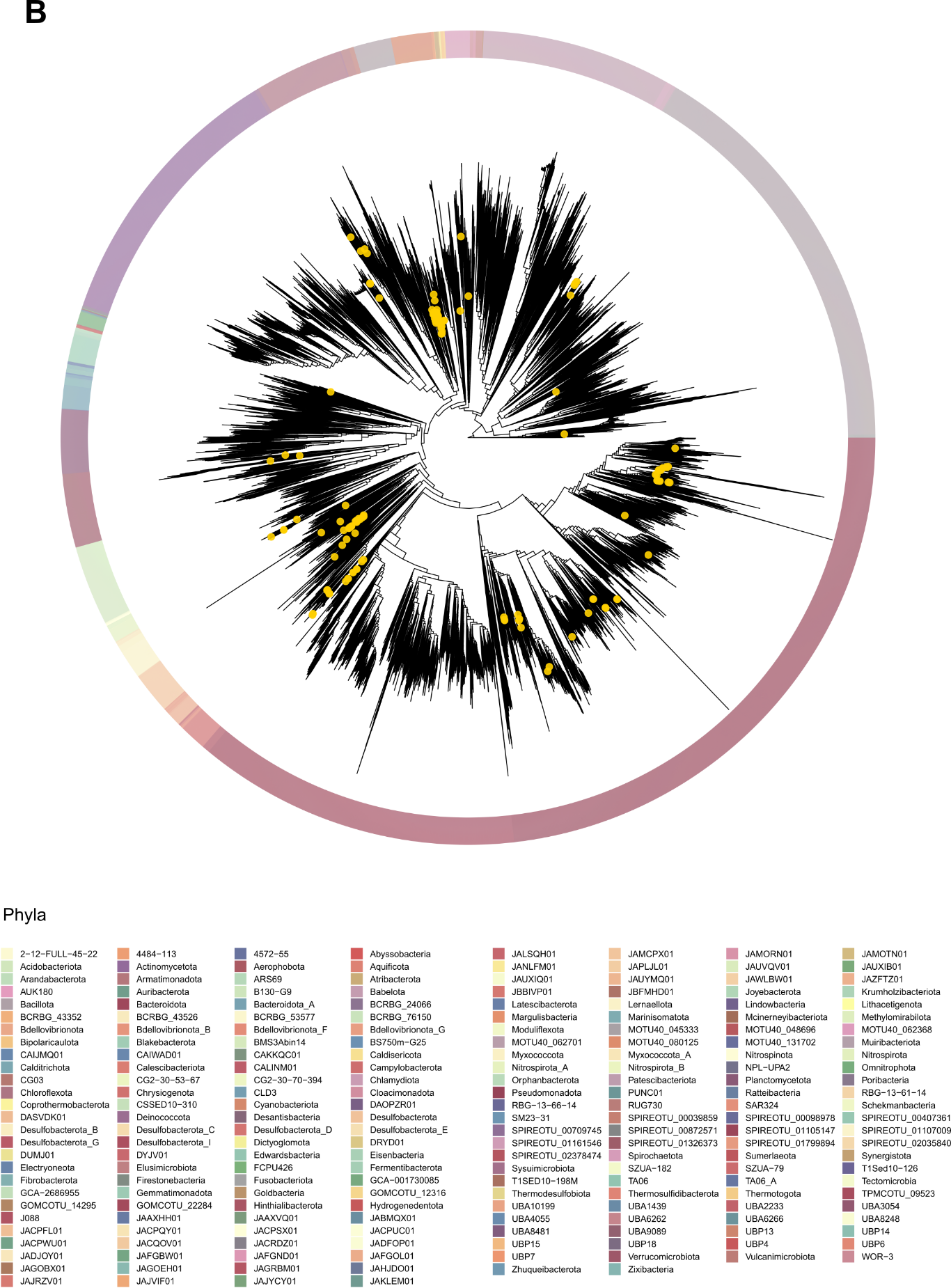
**

**Fig. S1. Phylogenetic distribution of putative CO dehydrogenases and putative Ni-CODH/ECH genes across 306,260 microbial genomes.**

(A) Archaeal and (B) bacterial genome trees based on the GTDB r226 backbone tree, encompassing 306,260 representative genomes from the GlobDB database (1). Outer rings indicate taxonomic affiliation at the phylum level. Yellow dots mark genomes predicted to encode a putative operon for Ni-CODH/ECH complex, defined as those harboring a *cooS* and [NiFe] Group 4 hydrogenase gene within 15 genes.


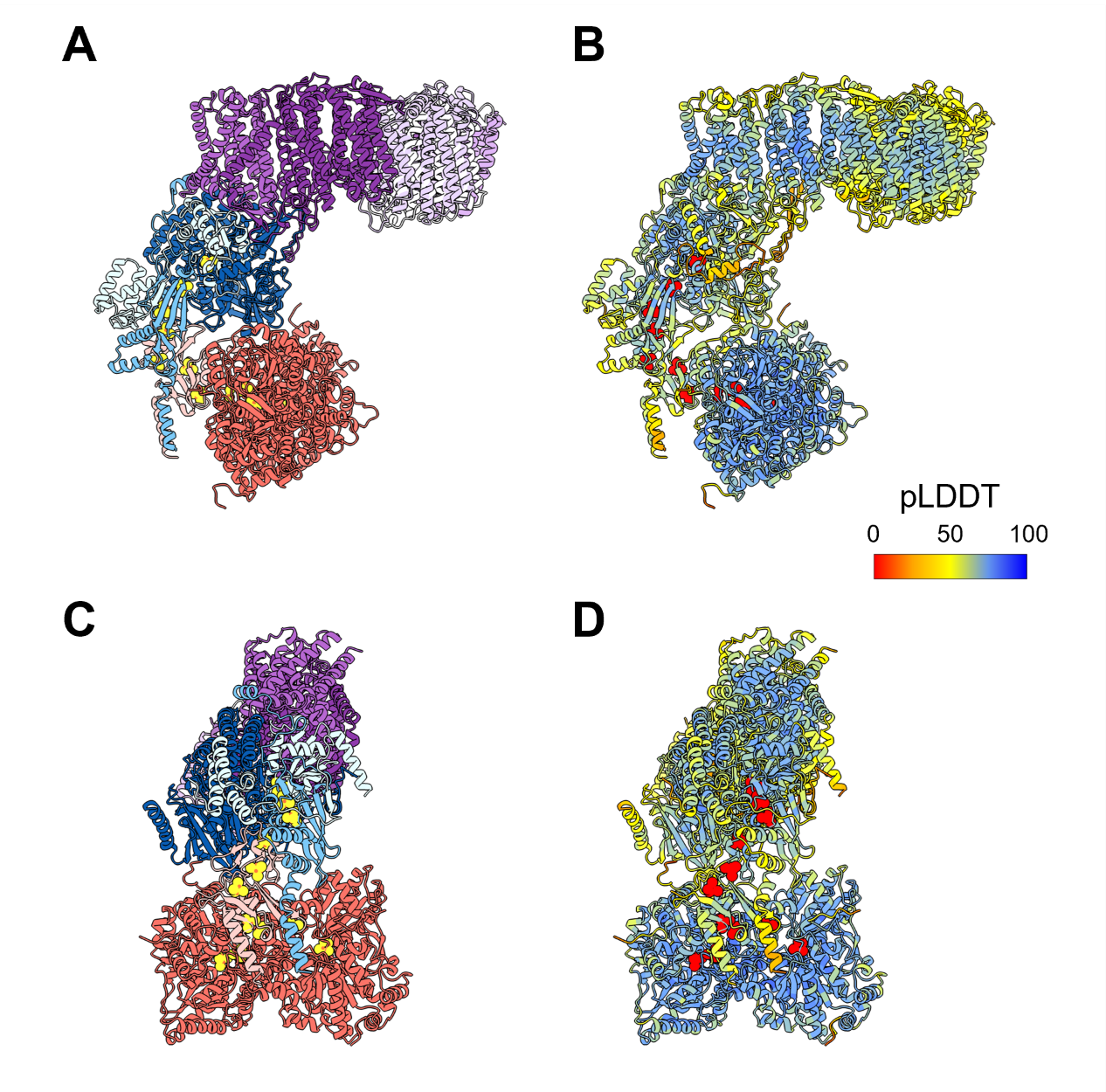


**Fig. S2 AlphaFold 3 prediction confidence of the Ni-CODH/ECH structure from Parageobacillus sp. G301.**(A, C) Structures colored by subunit to illustrate overall architecture. (B, D) The same structures colored by AlphaFold 3 prediction confidence, as assessed by the predicted local distance difference test (pLDDT). Cofactors are shown in red regardless of pLDDT values. AlphaFold 3 models generated in this study have been deposited in Zenodo (https://doi.org/10.5281/zenodo.18617366).


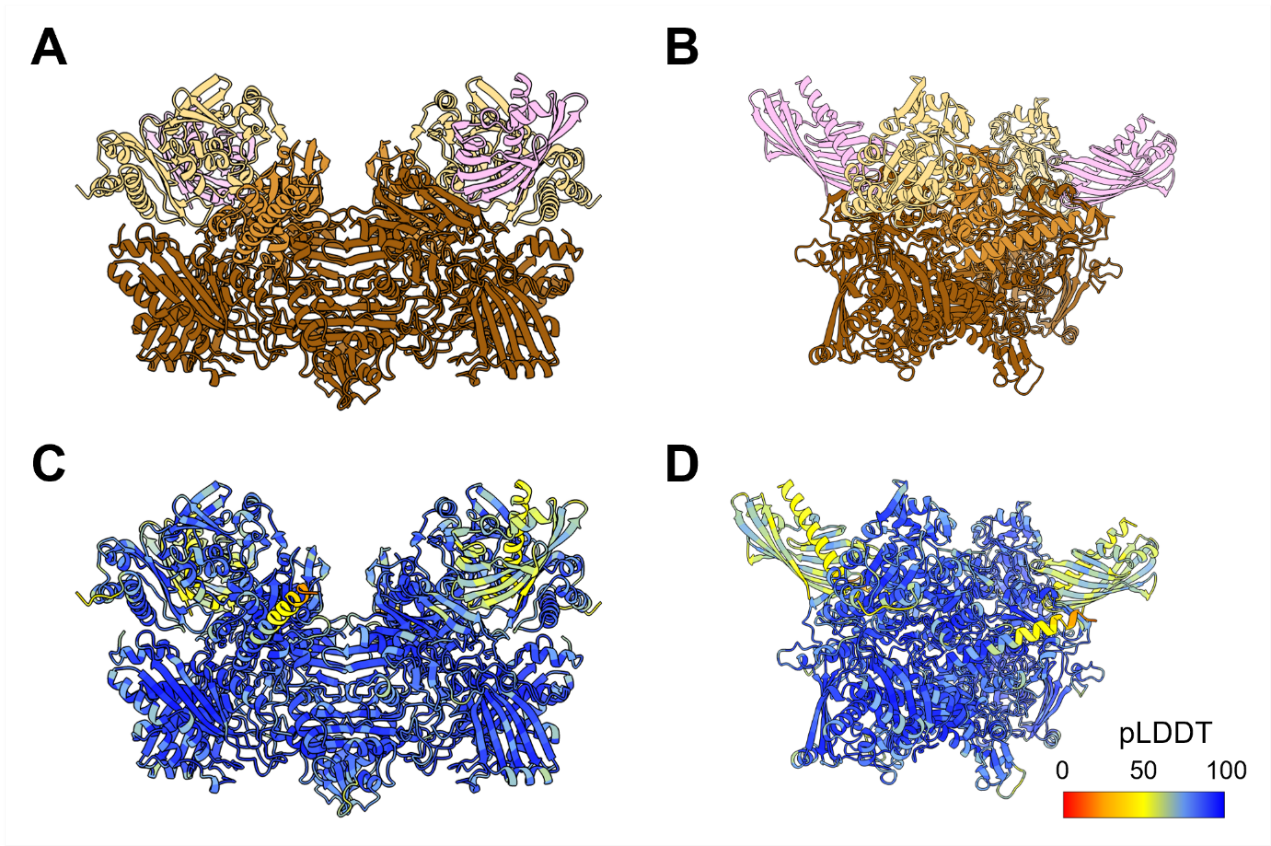


**Fig. S3. AlphaFold 3-predicted structures of the Mo-CODH (CoxMSLG) complex from *Parageobacillus* sp. G301.** (A, B) Structures colored by subunit to illustrate overall architecture, including the accessory subunit CoxG (pink). (C, D) The same structures colored by AlphaFold 3 prediction confidence, as assessed by the predicted local distance difference test (pLDDT). CoxG is predicted to associate with the CoxLMS core in a manner consistent with its proposed role in electron transfer. The AlphaFold 3 models are deposited in Zenodo (https://doi.org/10.5281/zenodo.18617366).


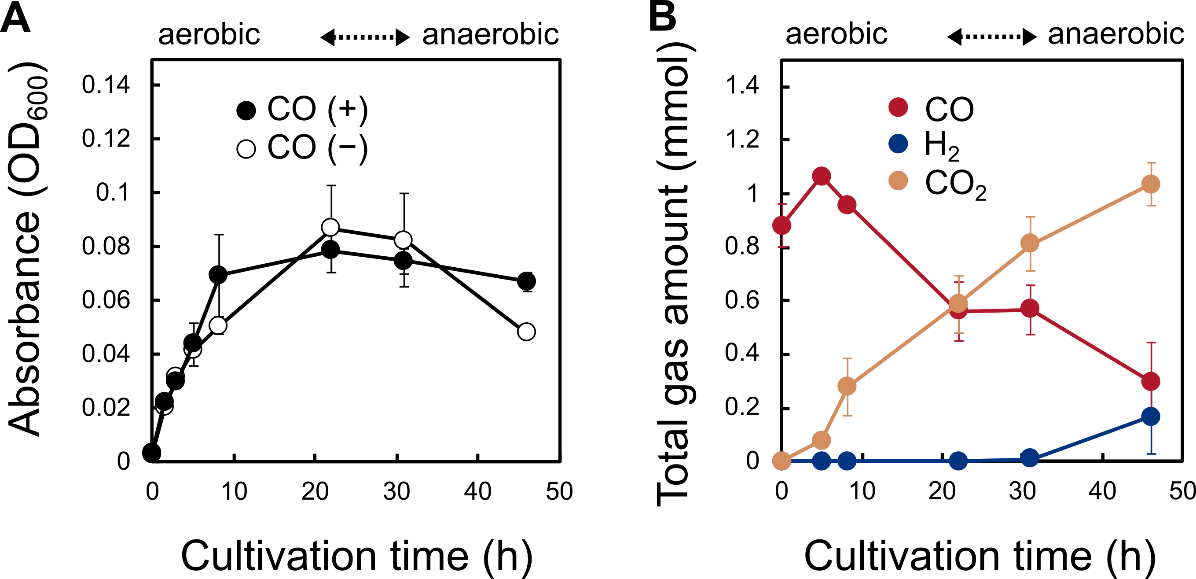


**Fig. S4. Growth and gas composition of *Parageobacillus* sp. G301 in the presence or absence of CO and under aerobic-microaerobic transition.** Cultures were grown in minimal medium supplemented with 5 mM sodium pyruvate under 15% O_2_ in the presence of CO (closed symbols) or in its absence (open symbols). (A) Growth curves (OD_600_). (B) Total gas amounts during the same incubations, with CO (red), CO_2_ (yellow), and H_2_ (blue) are shown. This experiment was conducted in sealed bottles, allowing a transition from aerobic to anaerobic conditions. Data represents means of three independent biological replicates, and error bars indicate the standard error of the mean.


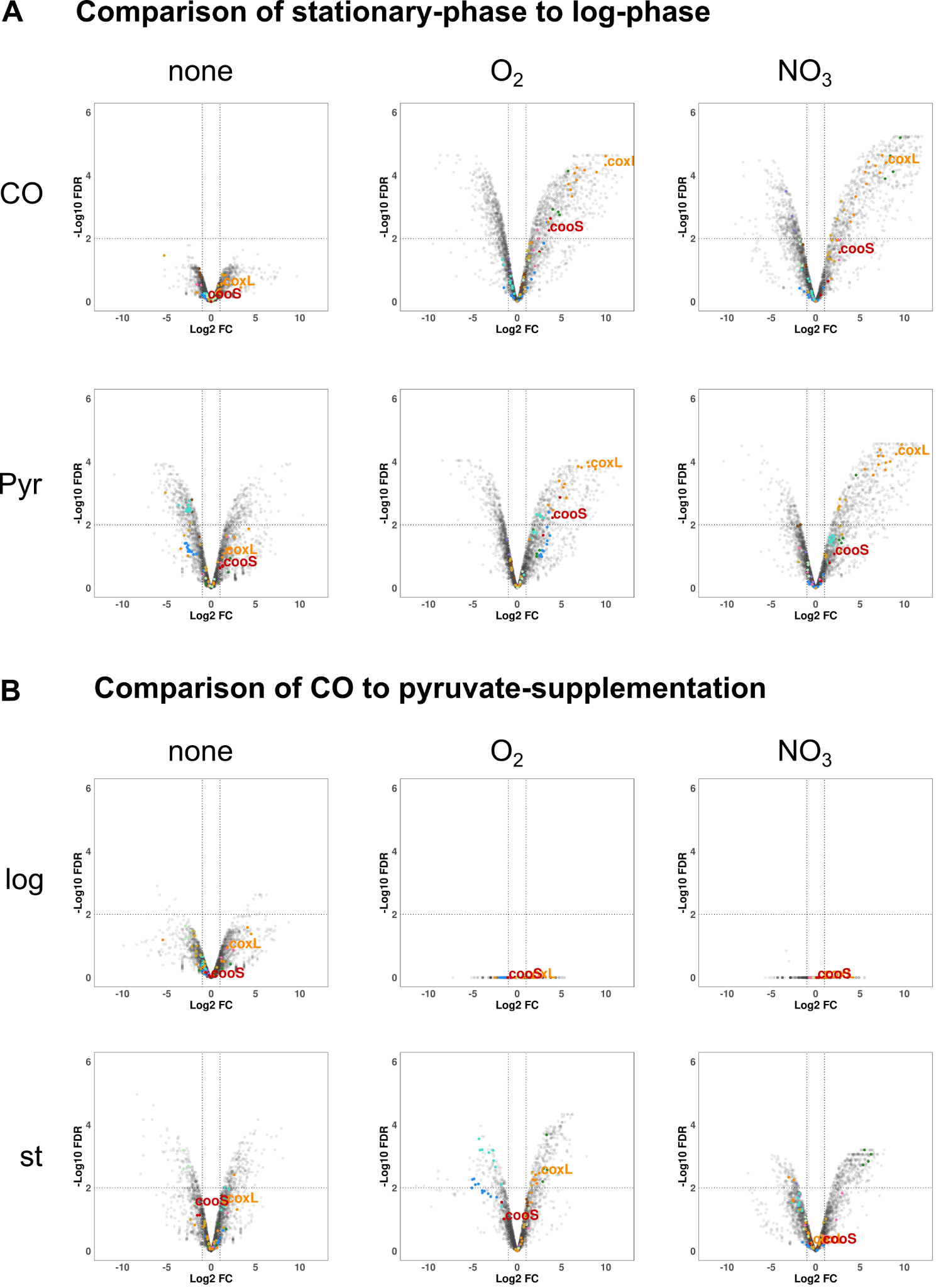


(legend on the next page)


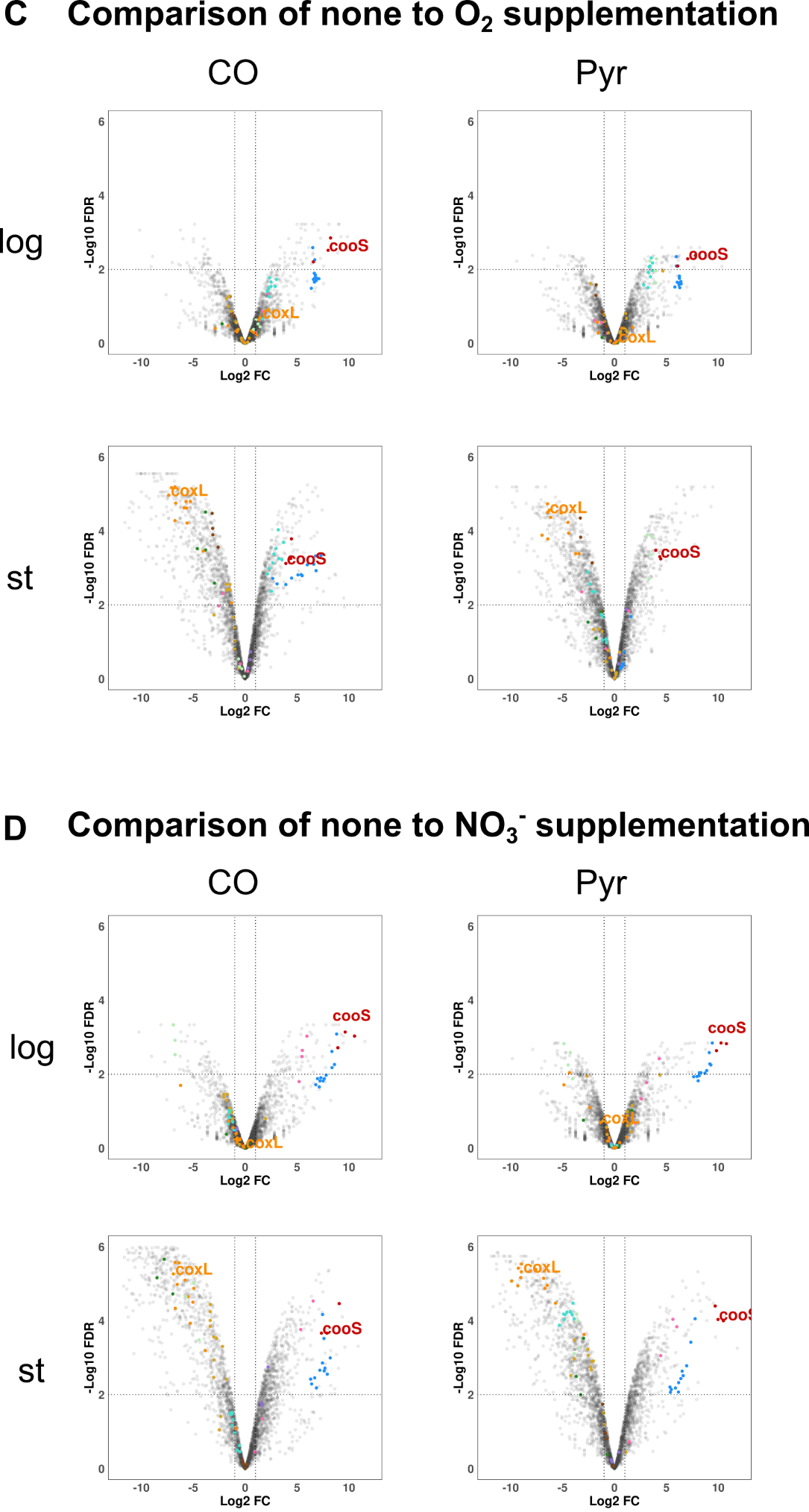


(legend on the next page)

**Fig. S5. Differential expression of *Parageobacillus* sp. G301 across substrates, electron acceptors, and growth phases.** Volcano plots show log_2_ fold change (x-axis) versus log_10_(FDR) (y-axis). Dotted vertical lines denote |log_2_FC| = 1 and the horizontal line denotes FDR = 0.01 (−log_10_FDR = 2). Selected markers are colored by functional group (see below), and *cooS* (Ni-CODH catalytic subunit) and *coxL* (Mo-CODH catalytic subunit) are labeled when present. Gray points are all other genes. (A) Comparisons of stationary-phase to log-phase transcriptomes. (B) Comparisons of CO to pyruvate supplementation as the available substrate. (C) Comparison of no external electron acceptor addition to O_2_ addition. (D) Comparison of no external electron acceptor addition to NO_3_^-^ addition. Colors are as follows (gene groups): Ni-CODH genes (*cooCSF*), red; energy-converting hydrogenase ECH genes (*hyfB–I*, *hycH/I*, *hypA/B*), blue; Mo-CODH genes (*moc/cox*, *ctaG*), orange; cytochrome bd oxidase (*cydABCD*), pink; nitrate reductase 1 (*narGHIJ*-1), dark green; nitrate reductase 2 (*narGHIJ*-2), light green; [NiFe]-hydrogenase (*hypA–F*, *hupF/hypC*, *hycI*, *hyaABC*), turquoise; Complex I (*nuoA–N*, *ndhF*), light brown; Complex II (*sdhABC*), purple; Complex IV (*ctaCDEF*), brown.

**
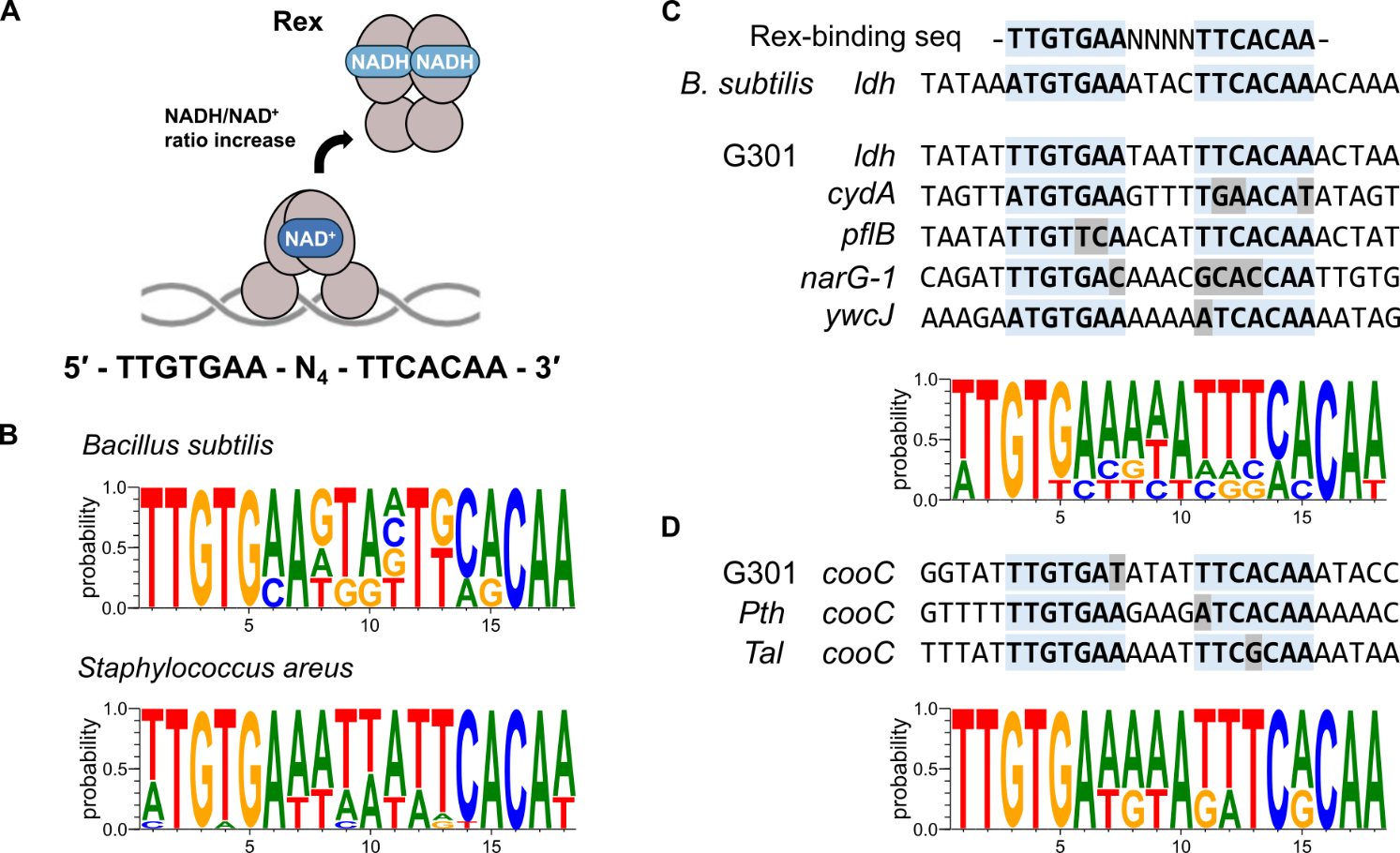
**

**Fig. S6 Predicted Rex-binding motifs upstream of redox-regulated genes in *Parageobacillus* sp. G301 and related species.**

(A) Schematic model of the redox-sensing transcriptional repressor Rex. Rex binds DNA as a homodimer when the intracellular NADH/NAD^+^ ratio is low and dissociates upon NADH binding, thereby derepressing target genes (11). The consensus Rex-binding sequence (5′-TTGTGAA-N_4_-TTCA CAA-3′) is shown below (12). (B) Experimentally validated Rex-binding motifs in *Bacillus subtilis* and *Staphylococcus aureus*, comprising four and fifteen sequences, respectively (13, 14). (C) Alignment of putative Rex-binding sites upstream of potential redox-regulated genes (*ldh*, *cydA*, *pflB*, *narG*-1, and *ywcJ*) in *Parageobacillus* sp. G301. (D) Predicted Rex-binding motifs upstream of the Ni-CODH/ECH gene (*cooC*) in *Parageobacillus* sp. G301, *P. thermoglucosidasius* (*pth*), and *T. altinsuensis* B1-1 (*tal*). The sequence logo indicates the conserved Rex-binding patterns. Nucleotides matching the consensus sequence are highlighted in light blue, and mismatches are shown in gray.


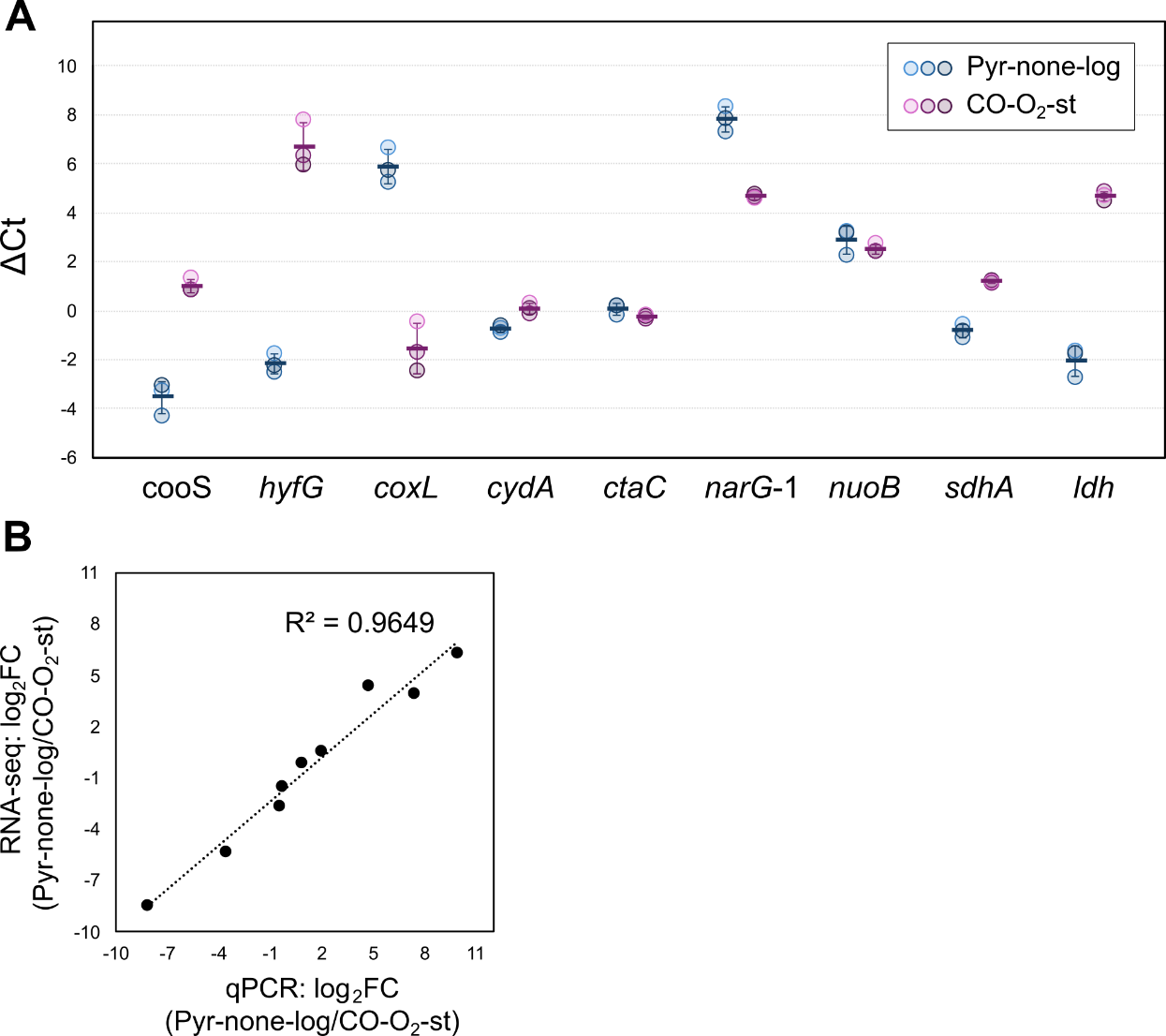


**Fig. S7. RT-qPCR validation of RNA-seq results in *Parageobacillus* sp. G301.** Relative transcript abundances of selected genes (*cooS*, *hyfG*, *coxL*, *cydA*, *ctaC*, *narG*-1, *nuoB*, *sdhA*, and *ldh*) were quantified by RT-qPCR under two conditions: pyruvate-grown cells without exogenous electron acceptors in the logarithmic phase (Pyr_none_log) and CO-grown cells with O_2_ in the stationary phase (CO_O_2__st). (A) ΔCt values normalized to the geometric mean of *gyrB* and *rpoB*. Points represent biological replicates (n = 3); horizontal bars indicate the mean, and error bars represent standard errors. (B) Correlation between log_2_ fold changes determined by RT-qPCR and RNA-seq (edgeR). Each point represents an individual gene. The dotted line indicates linear regression (*R*^2^ = 0.9649).


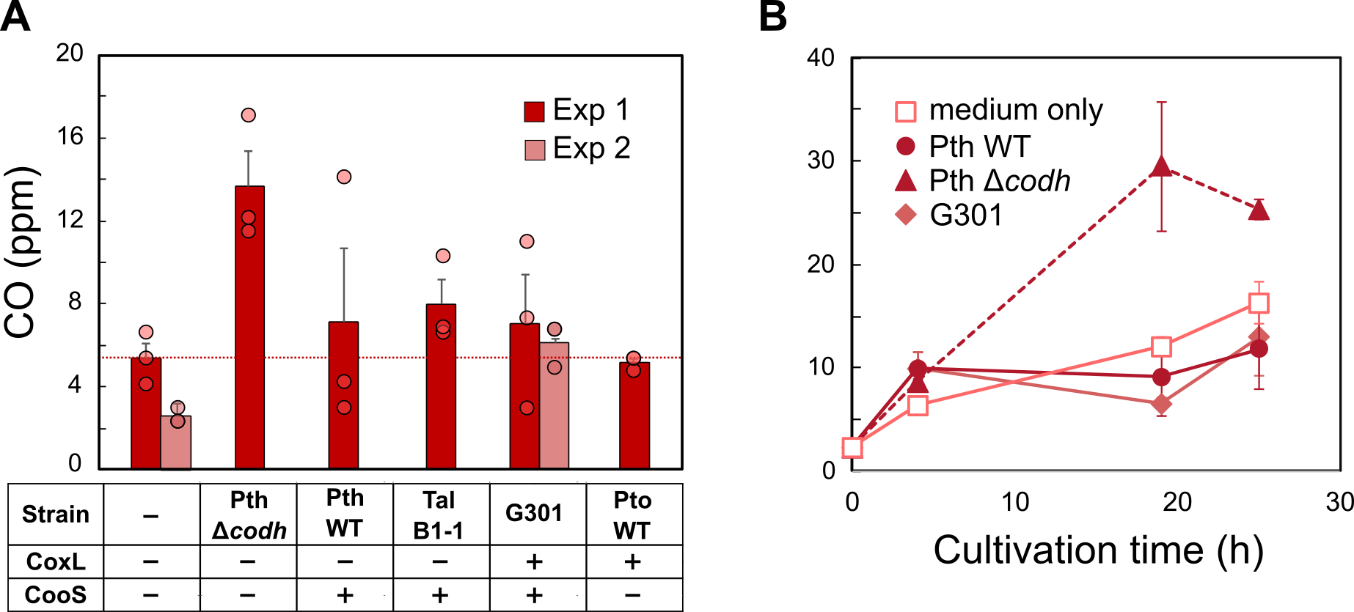


**Fig. S8 CO production by *Anoxybacillaceae* strains under anaerobic conditions.**

(A) The cells were cultured in TGP medium and CO concentrations measured at 4 h after inoculation. Two independent experiments (Exp 1 and Exp 2) are shown in dark and light red, respectively. For the Exp 2, only medium control and *Parageobacillus* sp. G301 were used to check reproductivity. Error bars represent standard deviations of biological replicates (n = 3). The dotted horizontal line indicates the CO level in the medium-only control. A table below the panel summarizes the presence (+) or absence (–) of Mo-CODH (CoxL) and Ni-CODH (CooS) in each strain. (B) Time course of CO accumulation in cultures of selected strains. CO concentrations were measured at 0, 4, 19, and 25 h. Note that each time point represents a different culture bottle. Strain abbreviations: Pto WT, *Parageobacillus toebii* NBRC 107807 (wild type); G301, *Parageobacillus* sp. G301; Tal B1-1, *Thermolongibacillus altinsuensis* B1-1; Pth WT, *Parageobacillus thermoglucosidasius* NBRC 107763 (wild type); Pth Δ*cooCSF*, CODH knockout mutant of *P. thermoglucosidasius* (Adachi et al., 2020). CO concentrations were measured using the CO detector tubes for these experiments (1LC; Gastec Co., Kanagawa, Japan). The error bars represent the standard error of the mean.

**References**

1. Speth DR, Pullen N, Aroney STN et al. GlobDB: a comprehensive species-dereplicated microbial genome resource. *Bioinform Adv* 2025;**5**:vbaf280. https://doi.org/10.1093/bioadv/vbaf280

2. Buchfink B, Reuter K, Drost HG. Sensitive protein alignments at tree-of-life scale using DIAMOND. *Nat Methods* 2021;**18**:366–8. https://doi.org/10.1038/s41592-021-01101-x

3. Søndergaard D, Pedersen CN, Greening C. HydDB: a web tool for hydrogenase classification and analysis. *Sci Rep* 2016;**6**:34212. https://doi.org/10.1038/srep34212

4. Yu G, Smith DK, Zhu H et al. ggtree: an R package for visualization and annotation of phylogenetic trees with their covariates and other associated data. *Methods Ecol Evol* 2017;**8**:28–36. https://doi.org/10.1111/2041-210X.12628

5. Wang LG, Lam TT, Xu S et al. Treeio: an R package for phylogenetic tree input and output with richly annotated and associated data. *Mol Biol Evol* 2020;**37**:599–603. https://doi.org/10.1093/molbev/msz240

6. Paradis E, Schliep K. ape 5.0: an environment for modern phylogenetics and evolutionary analyses in R. *Bioinformatics* 2019;**35**:526–8. https://doi.org/10.1093/bioinformatics/bty633

7. Xu S, Dai Z, Guo P et al. GgtreeExtra: compact visualization of richly annotated phylogenetic data. *Mol Biol Evol* 2021;**38**:4039–42. https://doi.org/10.1093/molbev/msab166

8. Wilke CO. cowplot: streamlined plot theme and plot annotations for ‘ggplot2’. The R Foundation; 2015. https://doi.org/10.32614/cran.package.cowplot

9. Zeileis A, Fisher JC, Hornik K et al. Colorspace: a toolbox for manipulating and assessing colors and palettes. *J Stat Softw* 2020;**96(1)**:1–49. https://doi.org/10.18637/jss.v096.i01

10. Cripps RE, Eley K, Leak DJ et al. Metabolic engineering of *Geobacillus thermoglucosidasius* for high yield ethanol production. *Metab Eng* 2009;**11**:398–408. https://doi.org/10.1016/j.ymben.2009.08.005

11. McLaughlin KJ, Strain-Damerell CM, Xie K et al. Structural basis for NADH/NAD⁺ redox sensing by a Rex family repressor. *Mol Cell* 2010;**38**:563–75. https://doi.org/10.1016/j.molcel.2010.05.006

12. Ravcheev DA, Li X, Latif H et al. Transcriptional regulation of central carbon and energy metabolism in bacteria by redox-responsive repressor Rex. *J Bacteriol* 2012;**194**:1145–57. https://doi.org/10.1128/JB.06412-11

13. Wang E, Bauer MC, Rogstam A et al. Structure and functional properties of the *Bacillus subtilis* transcriptional repressor Rex. *Mol Microbiol* 2008;**69**:466–78. https://doi.org/10.1111/j.1365-2958.2008.06295.x

14. Pagels M, Fuchs S, Pané-Farré J et al. Redox sensing by a Rex-family repressor is involved in the regulation of anaerobic gene expression in *Staphylococcus aureus*. *Mol Microbiol* 2010;**76**:1142–61. https://doi.org/10.1111/j.1365-2958.2010.07105.x
